# Triazole-based hybrid molecules in anticancer drug discovery: structural classes and mechanistic insights, 2014–2025

**DOI:** 10.3762/bjoc.22.94

**Published:** 2026-08-24

**Authors:** Meena Bhandari, Akshi Goyal, Deepak Yadav

**Affiliations:** 1 School of Basic and Applied Sciences, K.R Mangalam University, Gurugram, Haryana-122001, India; 2 Rajdhani College, University of Delhi, Delhi-110015, India

**Keywords:** anticancer agents, cancer cells, cytotoxicity, hybrids, 1,2,3-triazole, 1,2,4-triazole

## Abstract

Triazole-based hybrids have evolved into important pharmacophores in the search for anticancer drugs because of their varied biological profiles and structural adaptability. The versatility of triazole scaffolds in forming hybrid molecules with other compounds such as pyridines, coumarins, chalcones, pyrimidines, indoles, etc., has expanded the range of potential anticancer compounds. This review presents an overview of recent progress in the biological assessment of triazole-based hybrid compounds as potential anticancer agents, focusing on their structure, activity, and mechanisms of action. The findings outlined here underscore the therapeutic potential of triazole hybrids and lay the groundwork for designing future anticancer drug candidates.

## Introduction

Annually, almost 9 million individuals pass away from cancer-related causes [1]. Different types of carcinomas can develop because of abnormal cell division caused by internal as well as exterior causes, such as genetics, infections, medications, diet, and smoking [2–3]. Even though the incidence of this illness is increasing globally, an increasing percentage of cancer-related deaths are triggered by the disease's nearly crippling negative impact upon healthcare infrastructure, especially among poorer nations [4–5].

**Figure 1:**
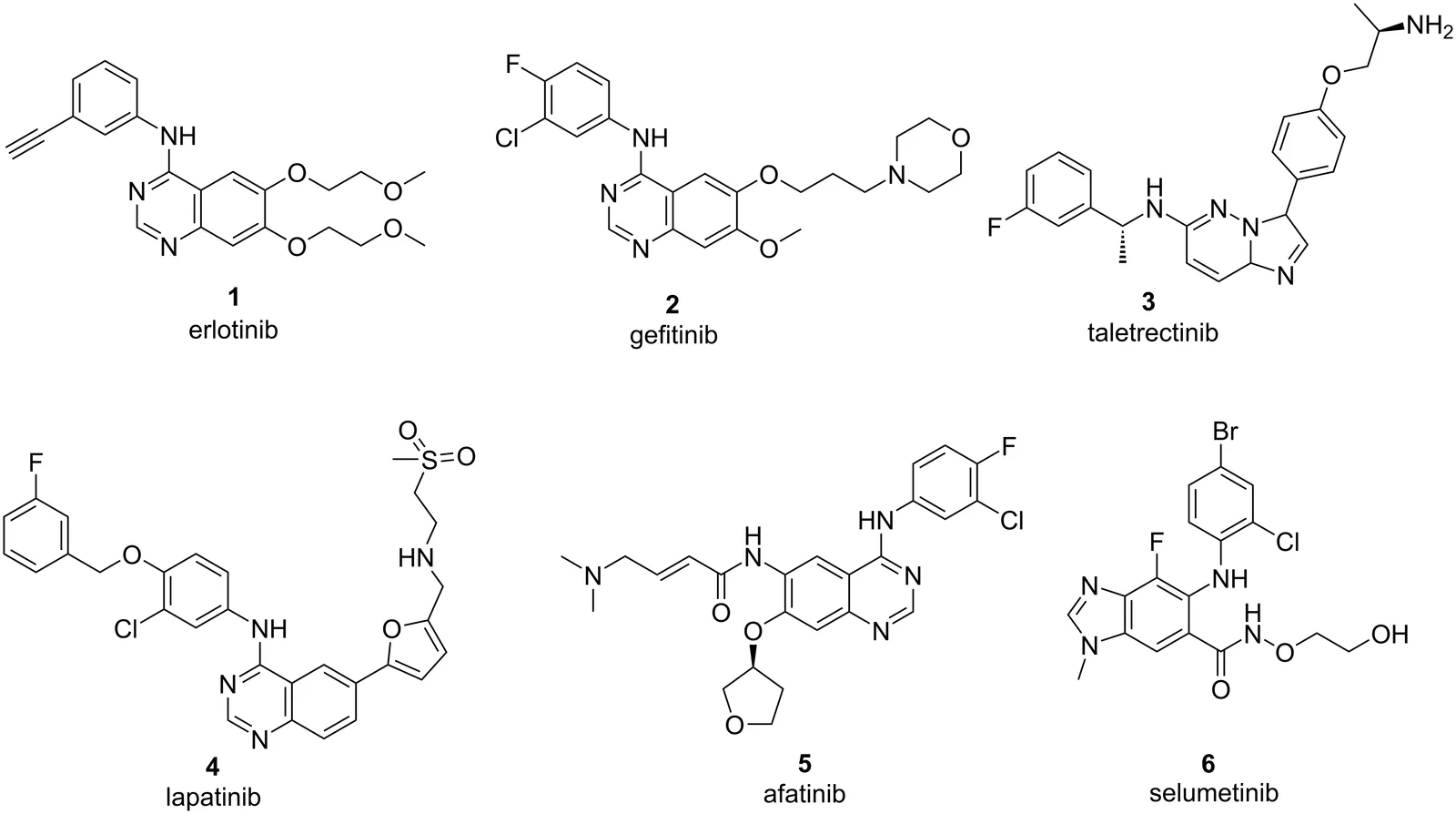
Some important examples of FDA approved anticancer drugs.

**Figure 2:**
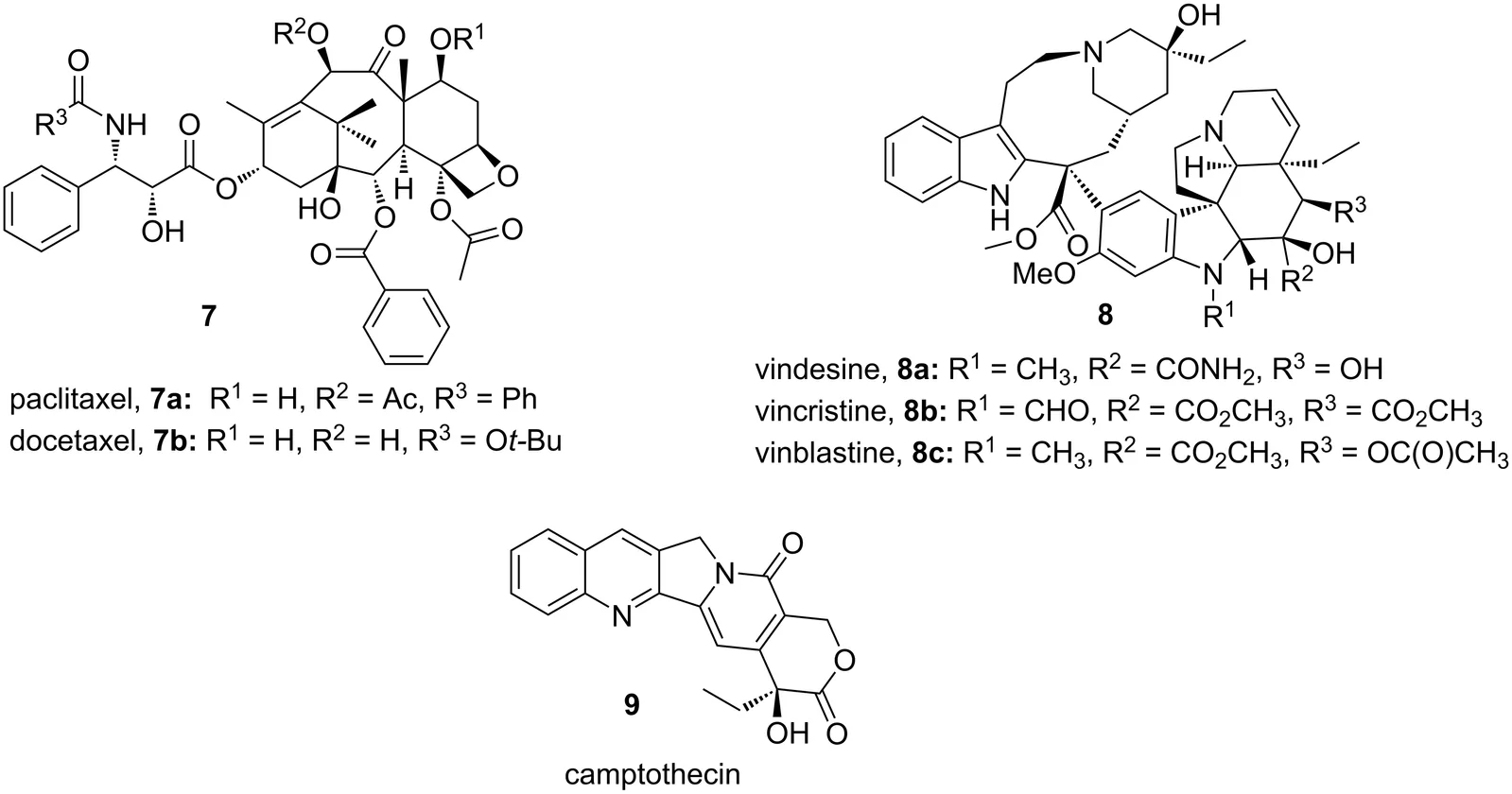
Some natural products based anticancer drugs.

Different combinatorial therapies such as immunotherapy, photodynamic therapy, hormone therapy, hyperthermia, stem cell transplantation, and surgery can be employed for the treatment of cancer, depending on the stage or type of cancer [6–8]. However, chemotherapy remains one of the most extensively employed treatment modalities for cancer, which employs a range of curative chemicals that selectively target rapidly dividing malignant cells while aiming to minimize damage to normal tissues [9–10]. Presently, more than hundred drugs have been approved for cancer management (Figure 1); but they are inefficient and show inadequate selectivity, that may give rise to adverse reactions [11–15]. Cancer-fighting medications produced by medicinal plants that are currently available include taxanes (docetaxel and paclitaxel), vinca alkaloids (vindesine, vincristine, and vinblastine), and camptothecin (Figure 2) [16–18]. Nevertheless, the limitations of existing chemotherapeutic agents highlight the need for the development of more potent and selective anticancer agents [19–20]. Therefore, the design of new effective anticancer drug remains one of the most demanding challenges in modern medicinal chemistry.

**Figure 3:**
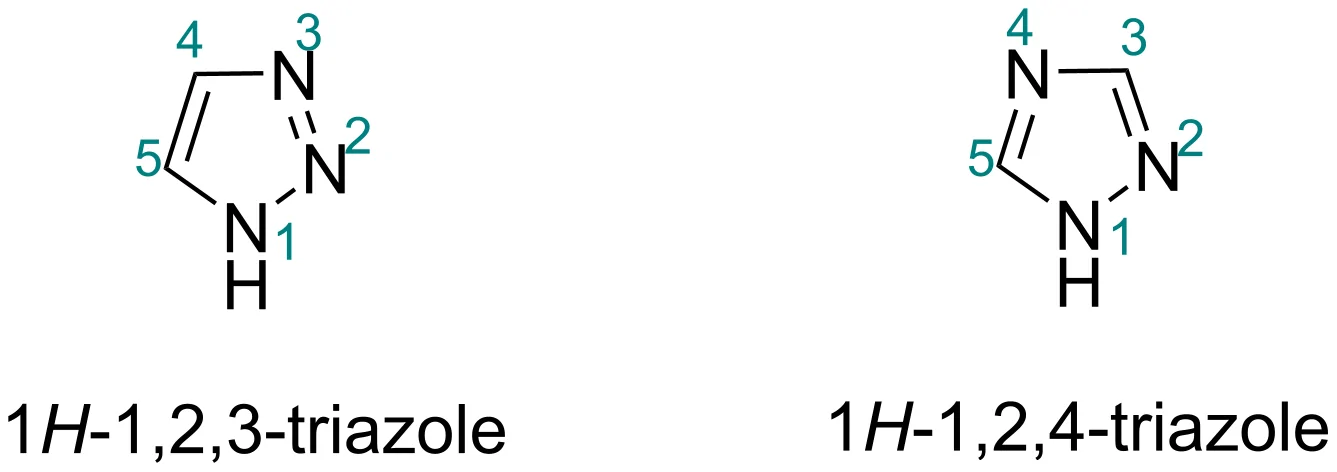
Chemical structures of isomeric triazole rings.

Owing to high metabolic stability, straightforward synthesis, and prospective to modulate biological processes, heterocyclic compounds – particularly triazoles – have attracted interest in therapeutic chemistry [21]. Triazoles are five-membered heterocycles with three nitrogen and two carbon atoms, existing in two isomeric forms, i.e., 1,2,3-triazole and 1,2,4-triazole (Figure 3) and several methods have been developed for their regioselective synthesis [22–24]. They have special qualities such as large dipole moments, π stacking, hydrogen bond formation ability, dissolution, and dipole–dipole interactions [25]. Their ability to form non-covalent interactions such as hydrogen bonds and van der Waals forces of attraction with various proteins, enzymes, and receptors that have high affinity towards these heterocycles make them highly promising in medical research [26–27].

**Figure 4:**
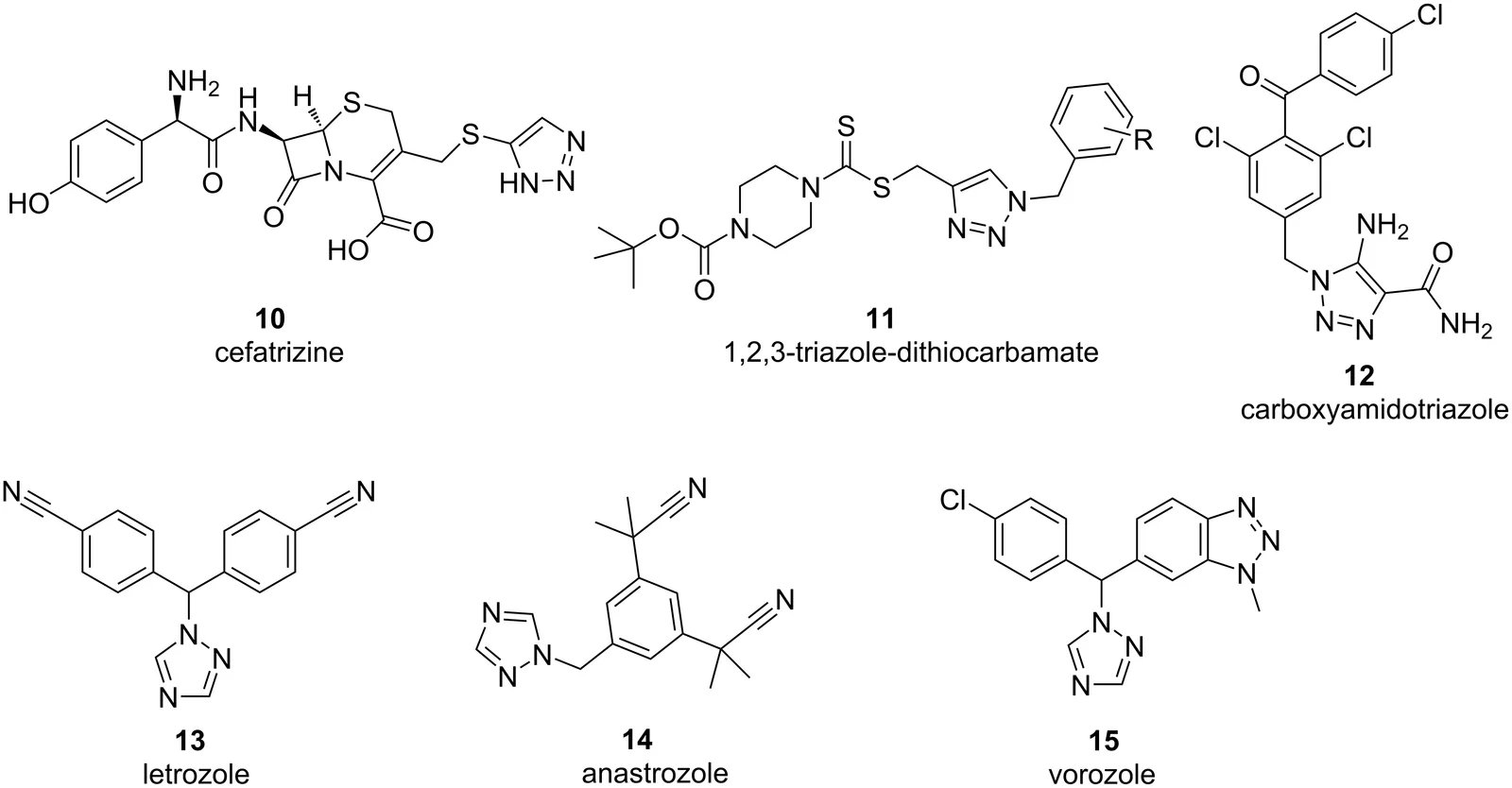
FDA approved drugs containing an 1,2,3- or 1,2,4-triazole moiety.

The 1,2,3-triazole scaffold has been strategically incorporated into several potential anticancer agents including cefatrizine (**10**), 1,2,3-triazole-dithiocarbamate **11**, and carboxyamidotriazole **12** (Figure 4) which have demonstrated efficacy across diverse cancer cell lines such as lung, prostate, colon, and breast. Though cefatrizine (**10**) showed no clinical development yet, 1,2,3-triazole-dithiocarbamate derivatives continue to be intriguing preclinical anticancer leads; nevertheless, no molecule from this class has yet progressed to human clinical trials. In phase II clinical trials, carboxyamidotriazole **12** has been used in conjunction with radiation therapy for the treatment of people suffering with supratentorial glioblastoma multiforme. In another randomized phase II trial, its efficacy towards metastatic kidney cancers was also explored. A random phase III trial was additionally undertaken to determine the effectiveness of the same triazole in managing people who have stage III or stage IV non-small cell lung cancer [28–29].

**Figure 5:**
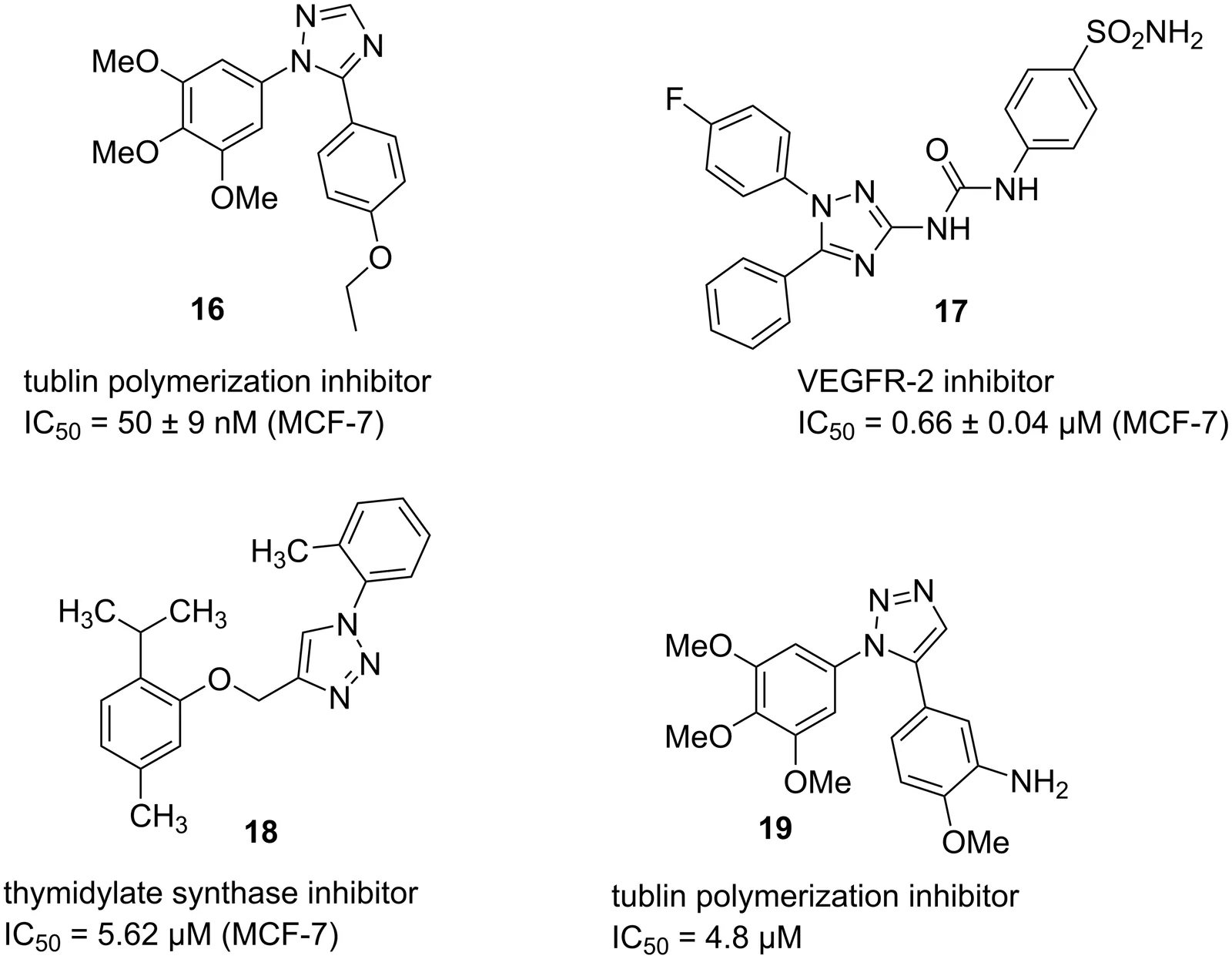
Anticancer compounds with an 1,2,3- or 1,2,4-triazole moiety.

Similarly, 1,2,4-triazole derivatives also exhibit considerable antineoplastic activity. At present, several clinically approved medications, including letrozole (**13**), anastrozole (**14**), and vorozole (**15**, Figure 4), which are members of the 1,2,4-triazole class, are widely employed in the management of estrogen-dependent breast cancer [30–32]. Beyond these established therapeutics, a variety of compounds with a triazole moiety (**16**–**19**, Figure 5) are acknowledged for their pharmacological properties, which include anticancer ability [33–36]. These examples demonstrate the potential of triazoles as anticancer agents. The strategic combination of the triazole core with other bioactive scaffolds has led to the development of hybrid molecules.

Hybrid pharmaceuticals are one type of molecules having “multiple targets" or "multiple ligands" exhibiting structural characteristics derived from two distinct parent molecules. Biologically active pharmacophores present in the hybrid systems can function independently at different pharmacological targets. Molecular hybrids have been developed to preserve their properties by combining biologically active substances that exhibit a two-fold activity mechanism. The coexistence of multiple pharmacophores within the same entity results in a pharmacological effectiveness that exceeds the cumulative effects of separate specific moieties potency. Combining two or more pharmacophores or producing hybrid molecules that incorporate two or more pharmacological constituents could serve as an efficient strategy to counteract cancer cells' susceptibility to medications [37]. These hybrid molecules offer multiple advantages over conventional anticancer agents. They can improve pharmacokinetic, pharmacodynamic, and physicochemical properties, minimize drug–drug interactions, and potentially overcome drug resistance [38]. Furthermore, it's vital to remember that hybrid medications typically defy Lipinski's and Veber's guidelines due to their large molecular mass and lipophilicity.

Triazole hybrids are appealing prospective molecules for drug discovery because they provide a multi-target approach to cancer treatment. Anticancer strategies are primarily aimed at disrupting the molecular systems that drive uncontrolled cell proliferation and survival. Among emerging therapeutic agents triazole-based hybrids have gained significant attention due to their ability to interfere with critical cellular processes. These hybrids can act on different targets that collectively disrupt tumor development and progression. They can inhibit topoisomerases, interfere with tublin polymerization, act as kinase inhibitors, trigger apoptosis through oxidative stress and cell cycle arrest, suppress angiogenesis, and interfere with DNA interactions [39–41].

In this review paper, research articles published in English language for the last twelve years were collected by using key words, triazoles, triazole hybrids and anticancer triazole compounds etc. A comprehensive analysis was carried out for the data obtained for the last 12 years (between 2014 and 2025). Only few classes of novel anticancer triazole hybrids were selected which elaborated the mechanism of action of synthesized compounds. This study used a qualitative strategy, a simple research framework with descriptive goals, and a systematic bibliographic approach to investigate the subject.

## Review

### Triazole-based molecular hybrids

**Figure 6:**
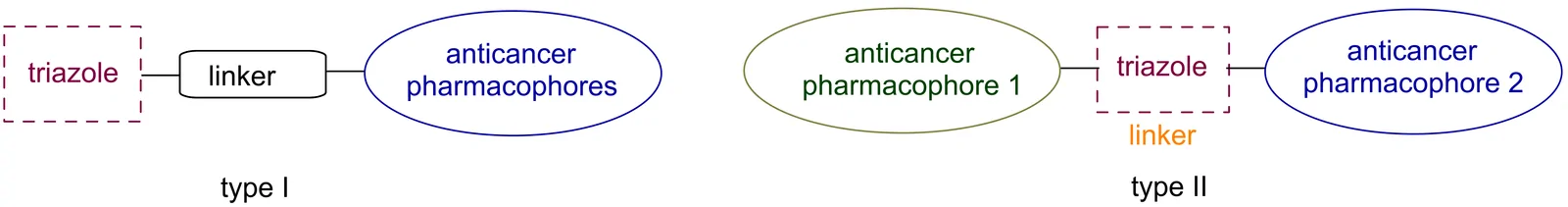
Types of triazole containing hybrids.

Advances in triazole-based molecular hybrids have revealed promising strategies for drug design and development. The hybrid molecules are created by combining two pharmacophores that are biologically active, with or without a suitable linker. In these hybrids the triazole moiety plays a crucial role in enhancing anticancer activities, improving selectivity, and overcoming resistance. It can act as bioactive core, pharmacophoric linker, or solubility and metabolic modulator [42–43]. Common pharmacophores used in hybridization include chalcone, quinoline, coumarin, indole, and pyridine moieties. Generally, triazole-containing hybrids fall into two major types (Figure 6); Type I: Different cytotoxic pharmacophores interact with the 1,2,3-triazole moiety; Type II: Two chemotherapeutic pharmacophores are connected via triazole linker [44].

These design strategies, including structural modification, incorporation of diverse pharmacophores, and exploration of different linkers, lead to the formation of numerous triazole hybrids with better efficacy across various pathological conditions, paving the way for further advancement in targeted drug therapies.

### Coumarin–triazole hybrids

**Figure 7:**
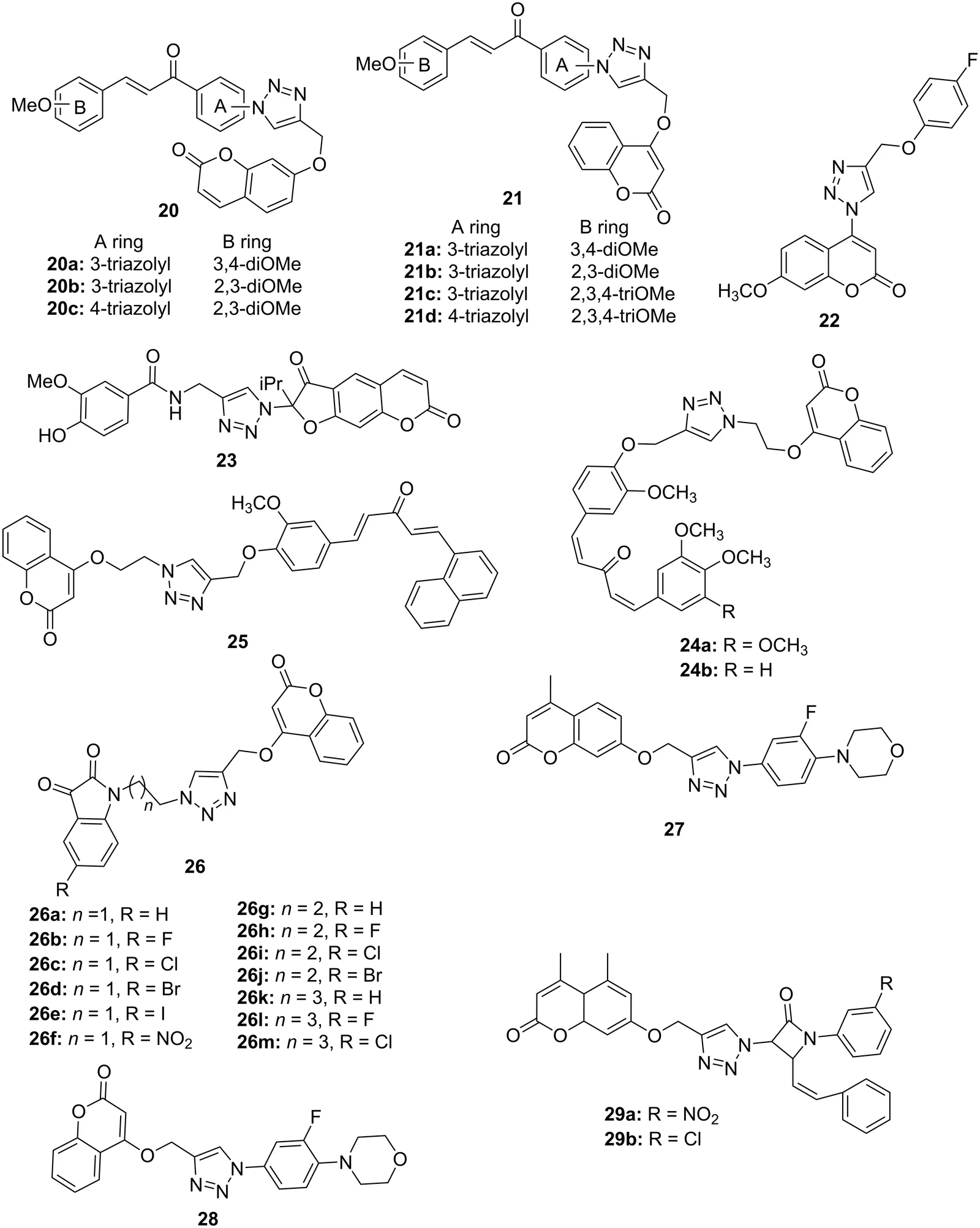
Structures of diverse coumarin–1,2,3-triazole hybrids **20**–**29**.

Although coumarin and 1,2,3-triazole derivatives individually demonstrated significant anticancer behavior, lead researchers have extensively explored hybrid compounds combining these pharmacophores for an enhanced and broader spectrum of action [45–46]. In recent years, coumarin–triazole hybrid molecules have emerged as promising candidates in drug discovery, owing to synergistic effects derived from both coumarin and triazole moieties. They exhibit diverse biological activities including anticancer, antioxidant, and antimicrobial [47–48]. These hybrid molecules show potential in combating drug resistance by acting on multiple targets, offering a superior alternative to combination therapy [49]. The synergistic effects of the combined pharmacophores contribute to their high therapeutic potential, making coumarin–triazole hybrids valuable lead molecules, recommended for studying in vivo behavior and potential drug advancement. For instance, chalcone–coumarin hybrids connected through a 1,2,3-triazole linker (**20**, **21**, Figure 7) have been investigated for their cytotoxic activity against various human cancer cell lines, including cholangiocarcinoma (HuCCA-1), human lung cancer (A549), hepatocellular carcinoma (HepG2), and malignant T-lymphoblastic (MOLT-3) cells [50]. Most of the compounds, with the exception of compound **20c**, demonstrated selective cytotoxic behavior for the MOLT-3 cell line, indicating good therapeutic potential. A structure–activity relationship (SAR) analysis showed that compounds **20b**, **20c**, and **21b** containing a triazole ring in 3- or 4-position of ring A and 2,3-dimethoxy groups on ring B, exhibited a strong activity against HepG2 cells, having a half-maximal inhibitory concentration (IC_50_) of 15.70 µM, 8.18 µM, and 4.26 µM, respectively. In contrast, compounds **21d** with trimethoxy substitution on ring B generally lost cytotoxicity towards HuCCA-1, HepG2 and A549 cells. However, compound **21c** reserved activity against HuCCA-1 with an IC_50_ of 6.13 µM. Compound **22** (Figure 7) 4-(1,2,3-triazol-1-yl)coumarin bearing a (4-fluorophenoxy)methyl substituent at the triazole C-4 exhibited potent antitumor activity against MCF-7, SW480, and A549 cell lines with IC_50_ values of 5.89 µM, 1.99 µM, and 0.52 µM, respectively [51]. Mechanistic investigations indicated that compound **22** inhibits cancer cell proliferation by promoting apoptosis and inducing G2/M cell-cycle arrest. SAR analysis revealed that a –CH_2_–O– linker at the C-4 position of the 1,2,3-triazole substantially increases cytotoxicity, whereas the presence of an analogous hydrogen acceptor substituent at the coumarin C-7 position favorably influences anticancer potency. The novel 2-(triazol-1-yl)dihydrofurocoumarin derivative **23** with 4-hydroxy-3-methoxybenzamidomethyl substituent at C-4 of the triazole ring was found active against leukemia cell lines including CEM-13 (acute lymphoblastic leukemia), MT-4 (membrane-type 4 T-cells), and U-937 (myeloid leukemia) and showed selectivity with cytotoxic dose (CTD_50_) values of 9.00 µM (CEM-13), 10.00 µM (MT-4), 8.00 µM (U-937) [52]. Also, this compound exhibited a strong binding affinity of −9.2 kcal/mol with PDE4B (phosphodiesterase-4B), which is better to the reference drug, rolipram (−8.3 kcal/mol), indicating its potential as an anticancer lead. The curcuminoid–coumarin hybrids tethered through a 1,2,3-triazole linker (**24a**,**b** and **25**, Figure 7) showed promising cytotoxicity towards THP-1, COLO-205, and HCT-116 cancer cell lines with IC_50_ values ranging from 0.82 to 9.95 µM but displayed no significant effect on PC-3 cells. Importantly, the two-carbon spacer flanked by coumarin and triazole units was found essential for the activity [53]. The isatin–coumarin hybrids **26a**–**m** (Figure 7) which exhibited potent activity, especially compound **26a**, showed IC_50_ values of 0.73 µM (THP-1), 3.45 µM (COLO-205), and 3.04 µM (HCT-116), along with strong tubulin polymerization inhibition (IC_50_ = 1.06 µM) [54]. Also, fluorine-substituted isatin derivative **26b** showed notable activity against THP-1 (IC_50_ = 1.99 µM). SAR analysis indicated that extending the linker or modifying the isatin ring reduces the cytotoxic potency. The coumarin–triazole hybrid **27** with 7-hydroxy-4-methylcoumarin connected to C-4 of the triazole ring (Figure 7) demonstrated strong cytotoxicity towards MCF-7 and HeLa cells lines, with IC_50_ values of 3.12 µM, and 2.77 µM, respectively, comparable to doxorubicin. Another derivative **28** (Figure 7), containing a 4-hydroxycoumarin moiety, also showed antiproliferative action for MCF-7 and Hela cells but both molecules have been found to be largely inactive against HEK-293 cells [55].

**Figure 8:**
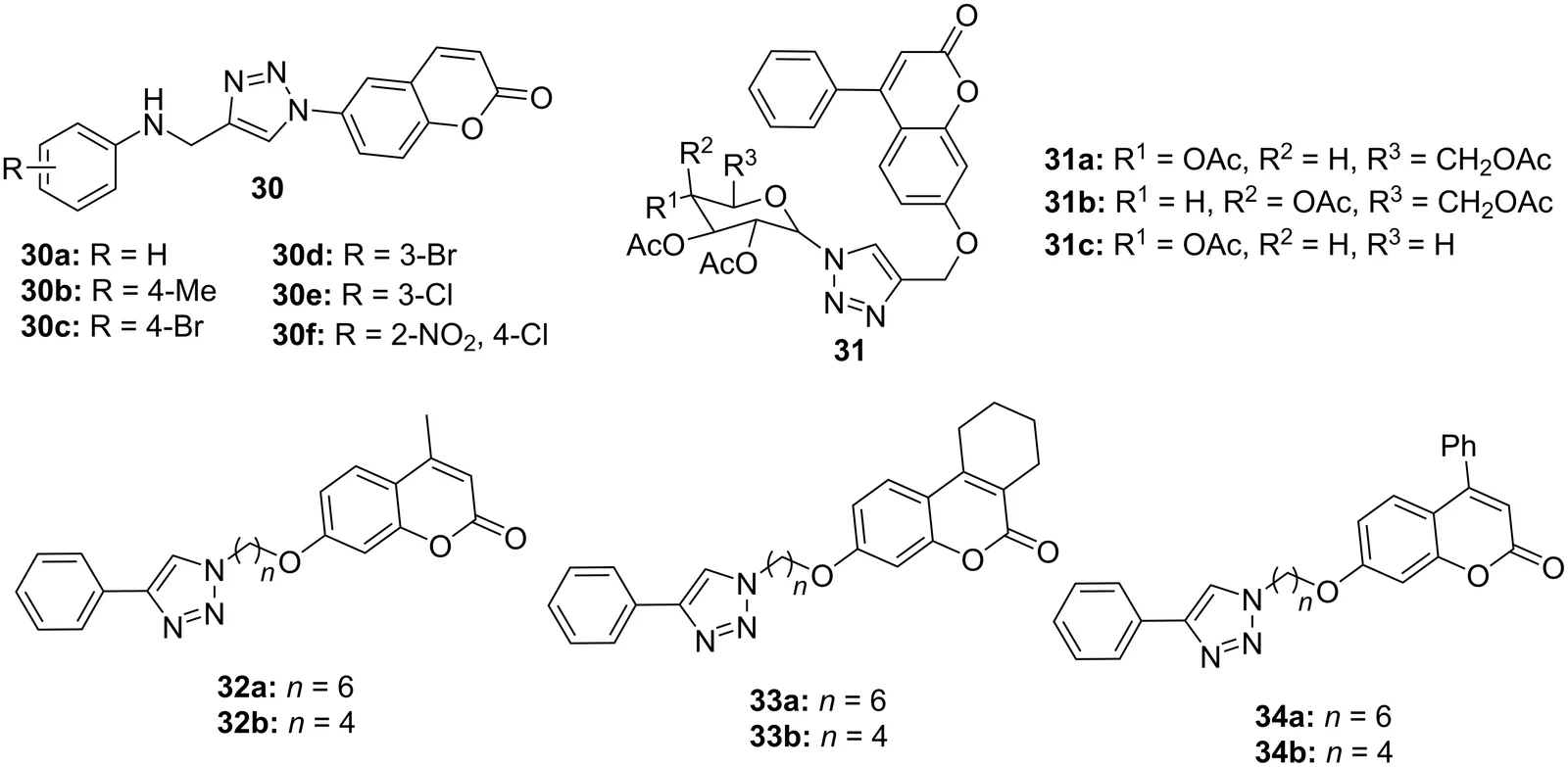
Structures of diverse coumarin–1,2,3-triazole hybrids **30**–**34**.

The coumarin-linked β-lactam–triazole hybrids **29a**,**b** (Figure 7) displayed modest action for MCF-7 having an IC_50_ concentration of 53.55 µM and 58.62 µM, respectively, and exhibited strong binding affinity to the estrogen receptor-α (ER-α). The presence of a nitro and chloro moiety at the C-3 position of the phenyl ring attached to N-1 of the β-lactam ring increased both potency and selectivity of these compounds for MCF-7 cancer cell lines [56]. The 4-anilinomethyl-1,2,3-triazole moiety tethered to the 6-position of coumarin **30a**–**f** (Figure 8) showed strong inhibitory activity (*K*_i_ < 100 nM) towards carbonic anhydrase isoforms IX and XIII which are involved in tumorigenesis [57]. The novel triazole–coumarin–glycosyl hybrids **31a**–**c** (Figure 8) have been evaluated for cytotoxic action towards different cancer cells. These compounds demonstrated promising growth inhibition with IC_50_ values ranging from 14.6–33.4 µM (Paca-2), 4.1–65.1 µM (MeI-501), 12.5–51.3 µM (PC-3), 9.9–14.5 µM (A-375) and 20.4–70.2 µM (Caco-2). Notably, compounds **31a** and **31c** are more effective against Paca-2 cells than doxorubicin (IC_50_ = 19.4 µM) [58]. Six new coumarin–triazole hybrids **32**–**34** (Figure 8) were studied for their action towards MCF-7 (breast cancer) and HeLa (cervical cancer) cells, among these candidates, **34b,** bearing a phenyl group, showed the strongest cytotoxicity. To enhance its delivery and efficacy, compound **34b** was encapsulated in poly(lactic-*co*-glycolic acid) (PGLA) nanoparticles. Importantly, the **34b**-loaded PGLA NPs showed significant higher anticancer activity than the free compound **34b**. The IC_50_ value dropped from 5.74 to 0.42 mg/mL against MCF-7 and from 1.32 to 0.77 mg/mL against Hela cells. These results pronounce the probability of use of coumarin–triazole derivatives, with nanoparticles-based delivery system, for enhanced cancer therapy [59].

**Figure 9:**
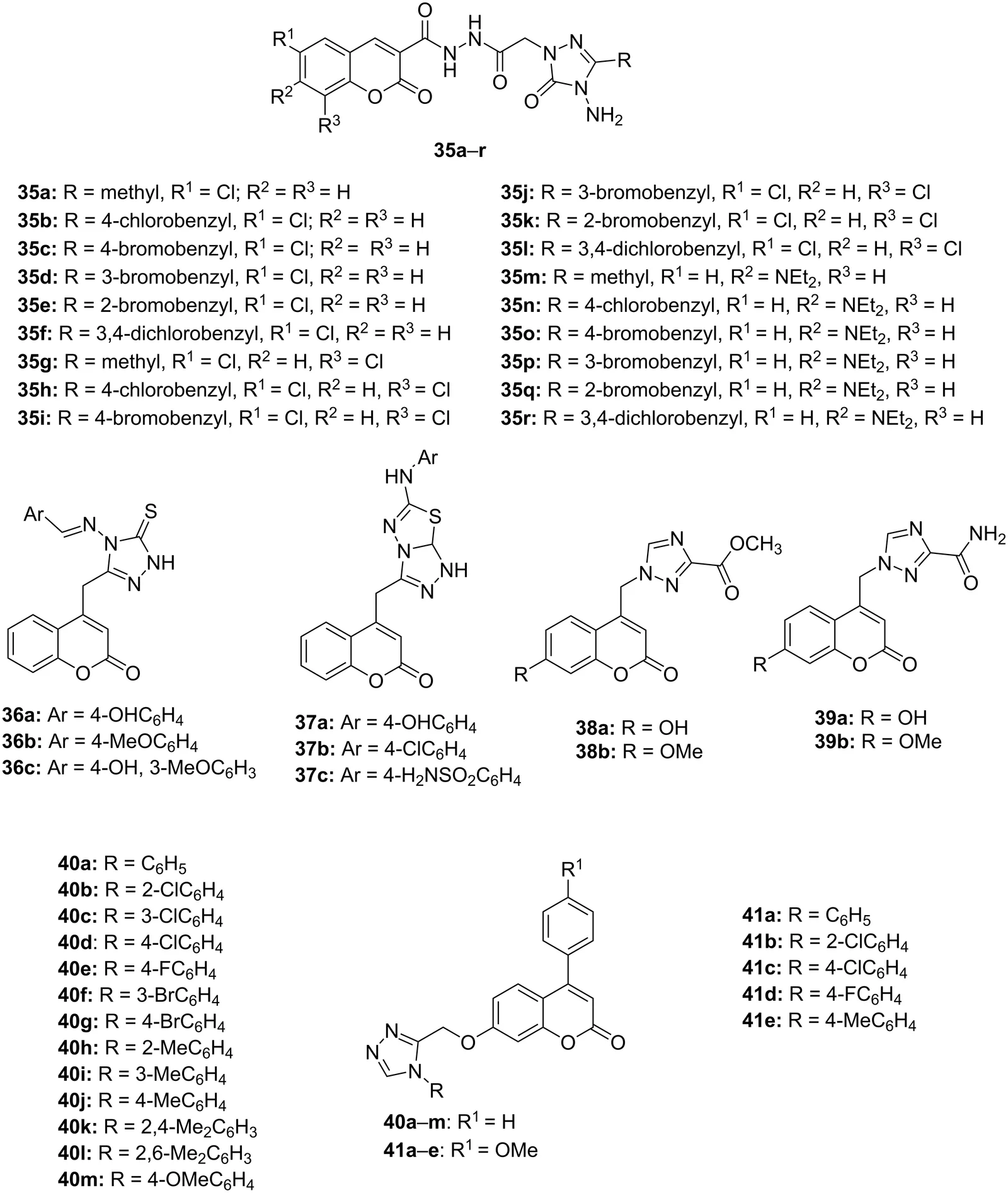
Structures of diverse coumarin–1,2,4-triazole hybrids **35**–**41**.

Beyond the 1,2,3-triazole-based system, 1,2,4-triazole hybrids have also garnered significant attention owing to their versatile pharmacological profiles and potent anticancer potential. Researchers have explored different synthetic methods and introduced structural modifications to enhance their therapeutic potential [60]. Here, several coumarin-1,2,4-triazole hybrids are discussed that have exhibited noteworthy anticancer activity. A series of *N*-acetyl carbohydrazide-linked 1,2,4-triazole–coumarin hybrids (**35a**–**r**, Figure 9) were evaluated for their cytotoxic activity against four human cancer cell lines: BT20 (breast carcinoma), A549 (lung carcinoma), SK-Mel-128 (melanoma), and DU-145 (prostate carcinoma) [61]. Their performance was compared with the standard chemotherapeutic agent cisplatin. These derivatives displayed anticancer action ranging from moderate to high efficacy across the tested cell lines. Specifically, compounds **35f, 35h–i**, **35k**, **35p**, and **35r** showed superior anticancer action towards the BT20 cell line, with cytotoxic concentration (CC_50_) values fluctuating between 3.6 and 13.9 µg/mL and selectivity indices between 2.4 and 5.2. Compound **35f** demonstrated enhanced activity against the DU-145 cell line (CC_50_ = 3.7 µg/mL, SI = 9.9), while compounds **35h** and **35i** were more active than cisplatin against A549 cells (CC_50_ = 7.5 and 8.1 µg/mL, SI = 4.2 and 3.3). However, none of the tested molecules surpassed cisplatin in efficacy against the SK-Mel-128 melanoma cell line. Novel coumarin derivatives connected to 1,2,4-triazole (**36a**–**c**, Figure 9) or 1,2,4-triazolo[3,4-*b*][1,3–4] thiadiazole groups (**37a**–**c**, Figure 9) via a methylene linker have been assessed as anticancer agents in human colon cancer cells (HCT116). Compounds **36c** and **37c** exhibit promising anticancer action having IC_50_ values of 4.363 µM and 2.656 µM, respectively. Molecular docking of active compounds proposes the interface with tyrosine kinase (CDK2) as a potential mechanism of action [62]. The methylene-bridged coumarin–1,2,4-triazole hybrids **38a**,**b** and **39a**,**b** (Figure 9) were assessed for their cytotoxic effects against several cancer cell lines, including HepG2 (liver), MCF-7 (breast), HeLa (cervical), and SW620 (colorectal) [63]. Among these, compound **39b**, containing a carboxamide group at C-3 of the triazole ring and a methoxy substituent at C-7 of the coumarin ring, displayed moderate cytotoxicity toward HeLa cells, with an IC_50_ value of 35.5 ± 13.5 µM.

The other compounds exhibited no significant effect on HeLa cells, displaying IC_50_ values greater than 100 µM, and none showed measurable effects on the remaining three cancer cell lines. Importantly, compound **39b** was non-toxic toward normal fibroblast cells, highlighting its selective action against cancer cells. A novel series of coumarin–triazole hybrids containing a C7-methoxy linker (**40a**–**m** and **41a**–**e**, Figure 9) were evaluated for anticancer activity against six human cancer cell lines: ASG, MGC-803, HCT-116, A549, HepG2, and HeLa. Among the synthesized candidates, **40d** exhibited the most potent activity against ASG and MGC-803 cells, with IC_50_ values of 2.63 ± 0.17 µM and 3.05 ± 0.29 µM, respectively. Compound **40c** showed strong inhibition toward HCT-116 cells (IC_50_ = 3.66 ± 0.24 µM). In addition, compound **40j** demonstrated cytotoxic effects against A549 (IC_50_ = 17.32 ± 0.63 µM) and HeLa (IC_50_ = 7.20 ± 0.54 µM) cells, while compound **40h** was found to be active against HepG2 cells (IC_50_ = 8.33 ± 0.27 µM) and compound **40m** showed selective toxicity towards Human gastric cancer (AGS) cells (IC_50_ = 9.00 ± 0.64 µM). The mechanistic evaluation showed that compound **40d** suppressed ASG cell proliferation by triggering apoptotic cell death and inducing cell cycle arrest at the G2/M phase. The replacement of the coumarin phenyl moiety with 4-methoxyphenyl (**41a**–**e**) was found to decrease the antiproliferative potency. Furthermore, compounds substituted with *para*-chloro and *para*-methyl groups demonstrated potent and wide-ranging anticancer efficacy across multiple cell lines [64]. These studies of coumarin-triazole hybrids represent their anticancer potential by combining the bioactive properties of both scaffolds.

### Indole–triazole hybrids

The indole ring is a highly prevalent, biologically active heterocyclic structure present in numerous natural compounds. It exhibits anticancer, antioxidant, anti-inflammatory, and antibacterial activity [65–66]. Due to diverse bioactivities, researchers have shown significant interest in designing and synthesizing indole-based hybrid molecules, such as indole–triazole, indole–coumarin, indole–isatin, and indole–pyrimidine to enhance therapeutic efficacy, target specificity, and reduce adverse effect [67]. Recent research has explored the potential of indole–triazole hybrids as anticancer agents. Indole and triazole units have been connected using several synthetic approaches, including click chemistry and various coupling reactions, to generate a diverse array of structurally distinct molecules for evaluating biological activity [68]. The flexible nature of indole–triazole conjugates allows for strategic modifications that can significantly influence their ADME profile as well as their interactions with biological targets. This adaptability makes them valuable scaffolds in medicinal chemistry, where fine tuning of their molecular architecture can lead to enhanced bioavailability, target specificity, and reduced toxicity.

**Figure 10:**
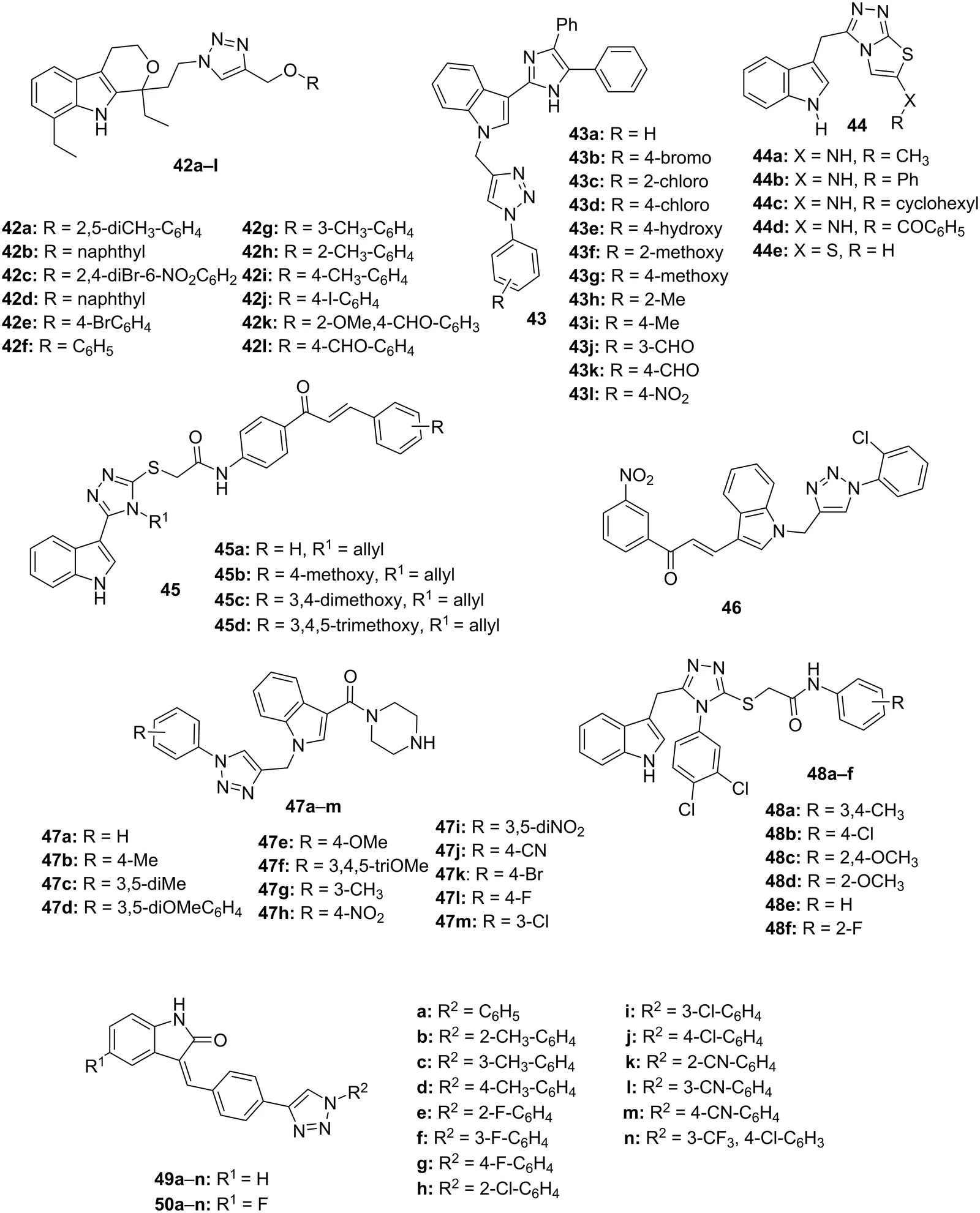
Structures of indole–triazole hybrids.

The cytotoxic study of etodolac-based derivatives containing a 1,2,3-triazole linkage (**42a**–**l**, Figure 10) against human lung cancer cells A549 showed that compounds **42e**, **42f**, **42h**, **42j**, and **42l** exhibited significant anticancer activity, surpassing the efficacy of the standard chemotherapeutic agent doxorubicin. Compound **42l** exhibited the greatest cytotoxic potency, showed an IC_50_ value of 3.29 ± 0.7 µM, closely followed by compound **42f**, which showed an IC_50_ of 3.65 ± 0.4 µM. The anticancer behavior of compound **42l** can be ascribed to inhibition of human topoisomerase II through specific molecular interactions [69]. 1,2,3-Triazole tangled indole analogues **43a–l** (Figure 10) have been tested for anticancer action with two human cancer cell lines MCF-7 and HepG2. The derivatives bearing 4-hydroxy, 4-methoxy, 2-methyl, and 3-acetyl substituents displayed superior cytotoxic action, with IC_50_ values lower than that of the standard chemotherapeutic agent, doxorubicin. In silico molecular docking analysis explained that the active molecules show robust binding affinities and key molecular connections through the active sites of aurora kinase-1 and DNA topoisomerase IIα, surpassing those of doxorubicin [70]. The molecular hybrids **44** containing 1,2,4-triazole and indole scaffolds were evaluated for their cytotoxic potential against the MCF-7 cell line. Out of synthesized candidates, **44d** and **44e** exhibited cytotoxicity with IC_50_ values of 17.67 ± 0.34 µM and 17.01 ± 0.53 µM, respectively. Compound **44e** demonstrated aromatase inhibition (IC_50_ = 0.026 µM), comparable to letrozole (IC_50_ = 0.024 µM) [71]. The study on anticancer activity of 1,2,4-triazole-tethered indolyl–chalcone scaffolds **45** (Figure 10) against NCI 60 cells demonstrated broad-spectrum antiproliferative effects of compounds **45a** and **45d,** having GI_50_ values from 3.69 to 20.40 µM and 0.29 to >100 µM, respectively [72]. Further examined was the inhibitory activity of selected hybrids against the epidermal growth factor receptor (EGFR) and c-MET (mesenchymal-epithelial transition factor) kinases. Compound **45b** displayed strong c-MET inhibition, with an IC_50_ value of 4.70 nM, comparable to the reference inhibitor foretinib (IC_50_ = 2.5 nM). Similarly, compound **45c** exhibited EGFR inhibition equivalent to that of erlotinib (IC_50_ = 0.052 µM) along with potent c-MET inhibitory activity (IC_50_ = 4.90 nM). These results highlight the promise of this scaffold as a potential dual kinase-targeted therapeutic agent. The indole–chalcone hybrid derivative functionalized with an *N*-substituted 1,2,3-triazole unit (**46**, Figure 10) exhibited strong anticancer activity against SiHa and SW620 cells having IC_50_ values of 67.99 and 48.96 µg/mL, respectively, while exhibiting minimal toxicity in normal human embryonic kidney HEK293 cells at comparable concentration. Mechanistic studies showed that compound **46** interacts with DNA through a non-covalent intercalative mechanism and contributes to its antiproliferative effects [73].

Lingaswamy et al. (2025) evaluated the anticancer potential of indole-piperazine-1,2,3-triazole derivatives **47a–m** (Figure 10) and found compounds **47d**, **47e**, and **47f** demonstrated superior cytotoxicity towards MCF-7, HCT-116 and HepG2 cancer cells, surpassing the efficacy of standard EGFR inhibitor erlotinib. Also, these compounds showed negligible toxicity towards normal breast epithelial cells (MCF-10A), demonstrating discriminatory anticancer action. Pharmacokinetics analysis showed that **47e** fully met major drug-likeness criteria, highlighting its promise as a selective EGFR-targeting anticancer agent [74]. The *N*-phenylacetamide derivatives incorporating an indole-1,2,4-triazole scaffold **48a–f** (Figure 10) were evaluated for cytotoxicity using doxorubicin and ellipticine as reference drugs. Compounds **48a**, **48c**, and **48f** exhibited remarkable anti-HepG2 activity, with cell-capability values of 11.72 ± 0.53, 18.92 ± 1.48, and 12.93 ± 0.55 µg/mL, respectively, demonstrating good to exceptional chemotherapeutic potential. Among them, the 3,4-dichloro-substituted derivative **48b** displayed pronounced cytotoxicity against HepG2 liver cancer cells, indicating its strong efficacy as a cellular-damage agent [75]. Indole-2-one-1,2,3-triazoles **49** and **50** (Figure 10) exhibited promising anti-angiogenesis activity as VEGFR-2 inhibitor and low toxicity towards human umbilical vein endothelial cells (HUVECs). Specifically, compound **49d** showed better kinase suppression (IC_50_ = 26.38 nM) than sunitinib (IC_50_ = 83.20 nM). Also, it effectively suppressed HT-29 and MKN-45 cancer cell growth. Molecular docking and molecular dynamics confirmed stable binding of **49d** to the VEGFR-2 active site [76].

**Figure 11:**
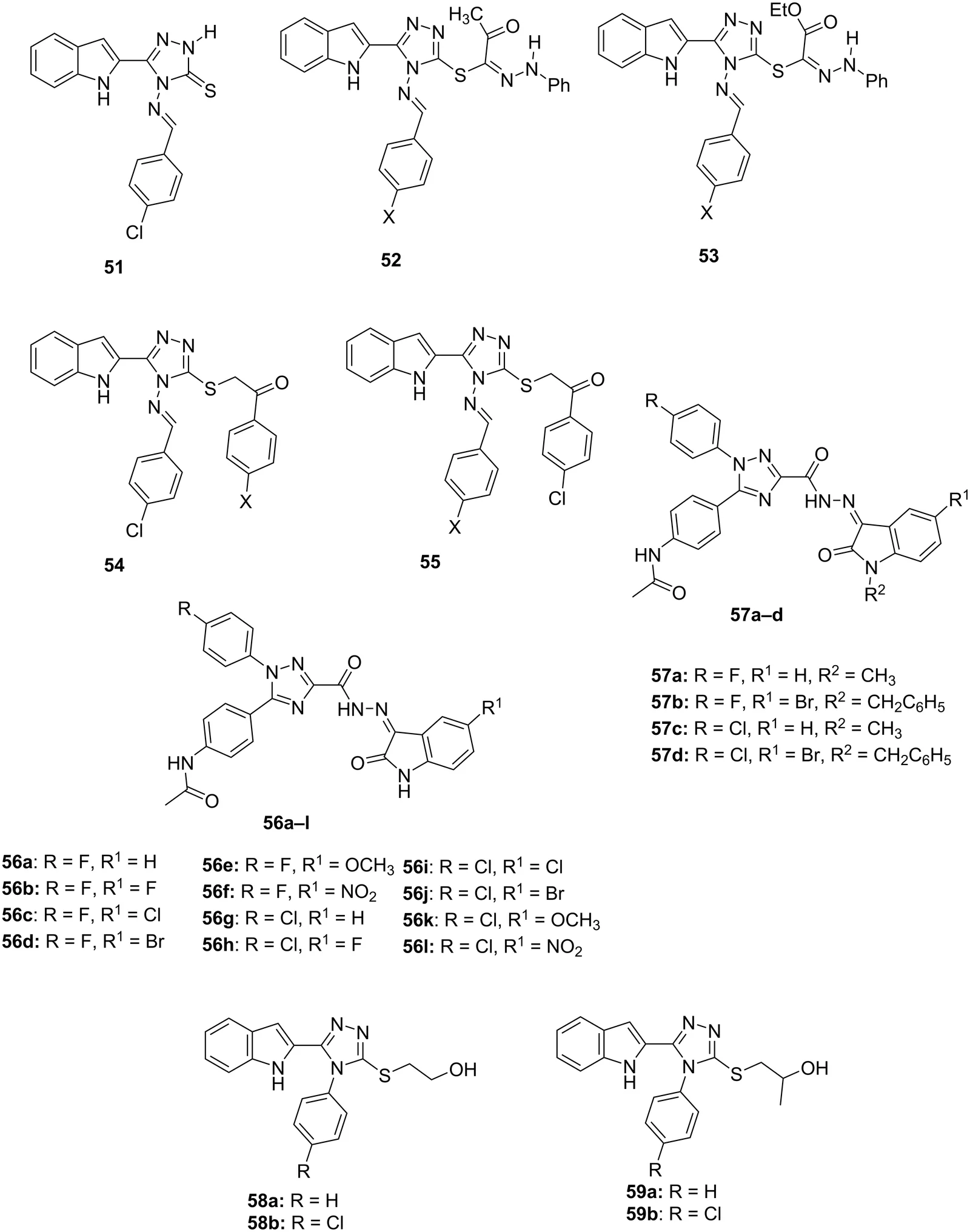
Structures of indoly–1,2,4-triazole hybrids and indolin-2-one-1,2,4-triazoles.

The indoly-1,2,4-triazole hybrids **51**–**55** (Figure 11) showed potent VEGFR-2 kinase inhibition and exhibited good antirenal cancer action. These derivatives exhibited superior VEGFR-2 inhibitory activity relative to the reference drug, sunitinib and revealed strong anticancer behaviour against CAKI-1 and A498 renal cancer cells [77]. Elsawi et al. (2024) developed 1,2,4-triazole-indolin-2-one derivatives **56** and **57** (Figure 11) as potential anti-hepatocellular and anti-pancreatic cancer agents, exhibiting VEGFR-2 inhibitory activity. These compounds demonstrated notable anticancer effects, with IC_50_ values ranging from 0.17 to 4.29 µM against PANC-1 cells and 0.58 to 4.49 µM against HepG2 cells. Several derivatives, specifically **56d**, **56e**, **56g**, **56k**, and **57c** showed potent VEGFR-2 inhibition, with IC_50_ values comparable to or exceeding that of the reference drug, sorafenib [78]. Indole-functionalized 1,2,4-triazole derivatives **58** and **59** (Figure 11) exhibited potent cytotoxicity against MCF-7 and HepG2 cells with IC_50_ values outperforming the standard drug erlotinib. Compound **59b** induced apoptosis caused cell cycle arrest at the G2/M and S phase in MCF-7 and HepG2 cells, respectively and demonstrated dual inhibition of PARP-1 and EGFR with IC_50_ values of 62.4 nM and 1.24 nM, surpassing erlotinib and olaparib. Furthermore, **59b** inhibited cancer cell proliferation by 66.7%, exceeding 65.7% efficacy [79]. These studies on indole–triazole hybrids have shown remarkable potential of these hybrids as anticancer candidates. Continued SAR studies and biological evaluation may pave the way for their advancement into potent therapeutic agents.

### Quinoline–triazole hybrids

In medicinal chemistry, quinoline is also recognized as highly valuable scaffold due to its presence in numerous bioactive natural products and its broad pharmacological profile, including anticancer, antimalarial, antibacterial, and anti-inflammatory effects [80–84]. Quinoline derivatives display anticancer activity through different mechanisms for instance apoptosis induction, cell cycle arrest, and angiogenesis inhibition [85–86]. However, some quinoline-containing drugs like chloroquine and fluoroquinolones are linked to cardiac side effects such as QT interval prolongation [87]. To overcome these limitations and enhance therapeutic potential, researchers are exploring quinoline-based hybrid molecules. The hybrid molecules containing quinoline and triazole rings have demonstrated significant potential across a wide range of biological activities [88].

**Figure 12:**
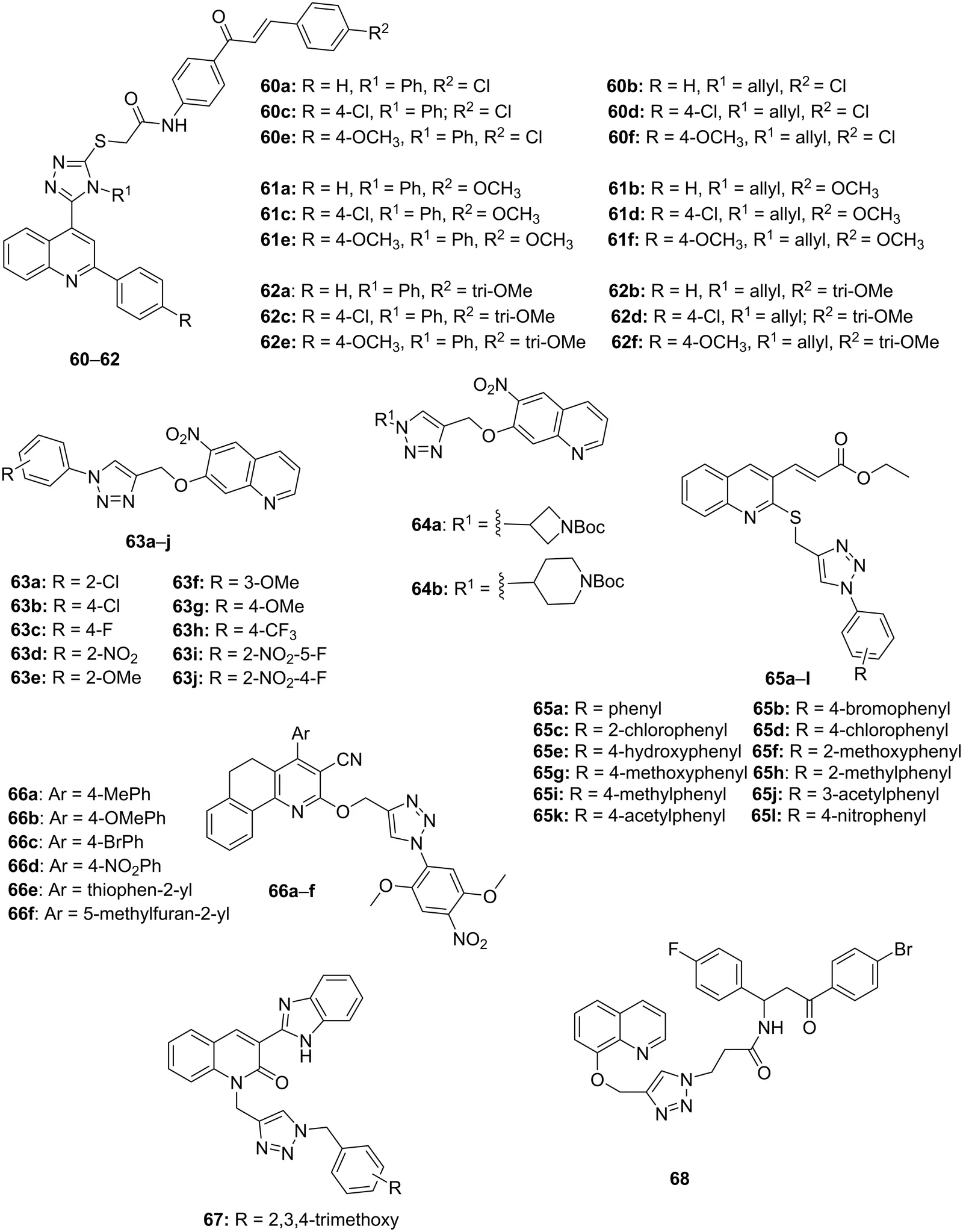
Structures of quinoline/quinolone–triazole hybrids **60**–**68**.

Mohassab et al. (2021) evaluated the anticancer potential of quinoline-chalone-1,2,4-triazole hybrids **60**–**62** (Figure 12) across a diverse panel of cancer cell lines, including several types of cancers such as leukemia, ovarian, renal, prostate, melanoma, lung, CNS, colon, and breast. Several compounds, particularly **60a**, **60b**, **62a**, **62b**, and **62d** exhibited significant antiproliferative effects and inhibited both EGFR and BRAF^V600E^ kinases. The most promising compound **62b** exhibited pronounced anticancer activity by promoting apoptosis and triggered G2/M phase cell cycle arrest. Also, the molecular docking analysis disclosed the significant binding connections with EGFR and BRAF^V600E^, with binding energies surpassing those of standard inhibitors like erlotinib and vemurafenib, respectively [89]. The quinolone–triazole hybrids **63** and **64** (Figure 12) exhibited moderate activity against MCF-7 breast cancer cells, while few of them displayed similar activity analogous to that of cisplatin. The specific substituents significantly influencing biological activity such as compounds with 2-chloro (**63a**) and 4-chloro (**63b**) substituents exhibit activity having IC_50_ values of 7.46 µM and 6.45 µM, respectively [90]. The new quinolone–1,2,3-triazole compounds **65a**–**l** (Figure 12) were assessed for anticancer activity towards HT-1080 and A-549 cells [91]. Here, many compounds exhibited weak to moderate cytotoxicity; however, derivatives, **65c**, **65d,** and **65j** bearing 2-chlorophenyl, 4-chlorophenyl and 3-acetylphenyl substituents, respectively demonstrated significant activity with IC_50_ values comparable to that of reference drug, doxorubicin (IC_50_ = 10.32 ± 1.32µM). El Malah et al. (2024) reported the anticancer potential of 1,2,3-triazolo-benzoquinoline-3-carbonitrile derivatives **66a**–**f** (Figure 12) against three human cancer cells HCT-116 (colon), HepG2 (liver), MCF-7 (breast) and a healthy cell line (BJ-1) and found compound **66b** demonstrated selective cytotoxicity against colon cancer (HCT-116), showing higher toxicity to cancer cells over normal ones. Against HepG2 liver cancer cells, compound **66c** exhibited potency surpassing the reference standard, doxorubicin [92].

**Figure 13:**
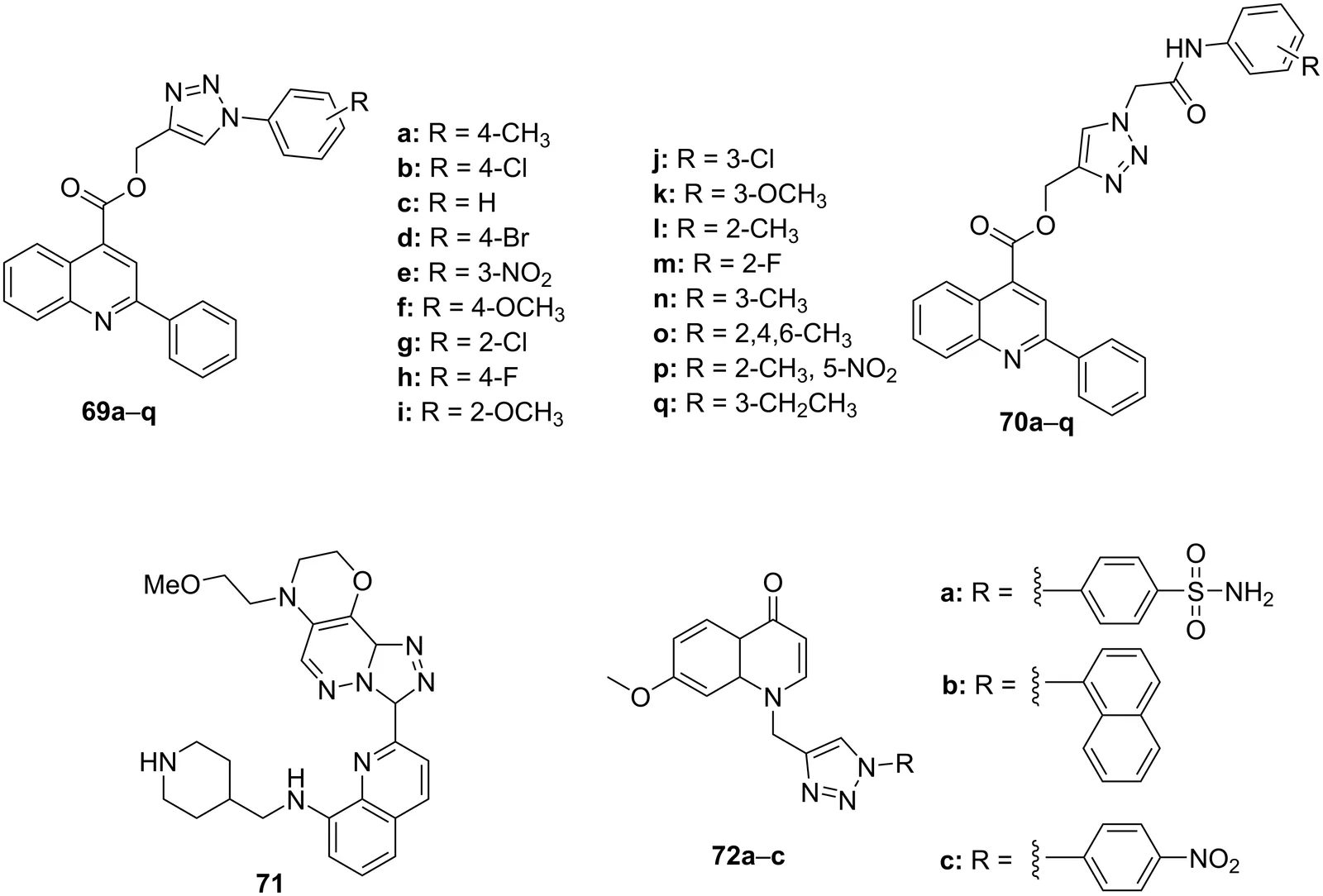
Structures of quinoline/quinolone–triazole hybrids **69**–**72**.

The 1,2,3-triazole compound incorporating a quinolone–benzimidazole framework **67** (Figure 12) exhibited notable anticancer activity towards a panel of NCI-60 human cancer cells, with favorable GI_50_, TGI, and LC_50_ values. This compound displayed a strong antiproliferative effect towards the BT-474 breast cancer cell-line, with an IC_50_ value of 0.59 ± 0.01 µM. The anticancer effect is induced primarily through ROS-mediated apoptosis in cancer cells [93]. The novel quinoline-triazole-linked peptidomimetic scaffolds **68** (Figure 12) showed inhibitory properties for CDK2, a protein implicated in various malignancies. An in vitro cytotoxicity assay against MCF-7 demonstrated that **68** repressed cell propagation with an IC_50_ value of 8 µM, highlighting its anticancer potential [94]. The two distinct quinolone–triazole hybrids **69** and **70** (Figure 13) were assessed for antiproliferative activity towards MCF-7 breast cancer cells. Among these synthesized hybrids, several derivatives such as **69h, 69i, 69l** and **69n** have exhibited promising antiproliferative action with IC_50_ values of 0.39–3.65 µg/mL, higher than the reference drug doxorubicin (IC_50_ = 3.69 µg/mL). Molecular docking analysis demonstrated powerful binding to the CDK2 enzyme with affinities ranging between −9.7 to −10 kcal/mol, emphasizing on the anticancer efficacy [95]. The novel quinolone conjugated with a triazolo[4,3-*b*]pyridazine moiety **71** (Figure 13) demonstrated antiproliferative activity across multiple cancer cell lines with GI_50_ values from 1.48–25.4 μM. The hybrid acts as an PIM-1/PIM-3 kinase inhibitor, exhibited IC_50_ values of 7 nM/70 nM, respectively [96]. The study on the anticancer potential of 7-methoxyquinoline–1,2,3-triazole hybrids **72** (Figure 13) across multiple cancer cell lines such as breast (BT-20, MDA-MB231, MCF-7) and colon (HT-29) cancers revealed that compounds **72a** and **72b** showed cytotoxicity, with IC_50_ values of 4.49 ± 0.68 µM and 19.05 ± 1.58 µM in BT20 and HT29 cells, respectively. In addition, interaction of MDA-MB-231 cells with **72c** resulted in pronounced nuclear morphological alteration, indicating their potential to act via apoptotic pathways [97]. These studies on quinolone–triazole hybrids have demonstrated their significant potential as anticancer agents. Their ability to act through multiple mechanisms and structural adaptability makes them a valuable scaffold for developing novel and effective anticancer therapeutics.

### Chalcone–triazole hybrids

These hybrid molecules integrate two pharmacological active components: chalcones, which contain an α,β-unsaturated carbonyl group, known to show interaction with various cellular targets involved in cancer progression, and 1,2,3-triazoles, which are recognized for their strong hydrogen bonding ability and metabolic stability. Together, these functionalities work synergistically to produce compounds with enhanced anticancer potential [98–100].

**Figure 14:**
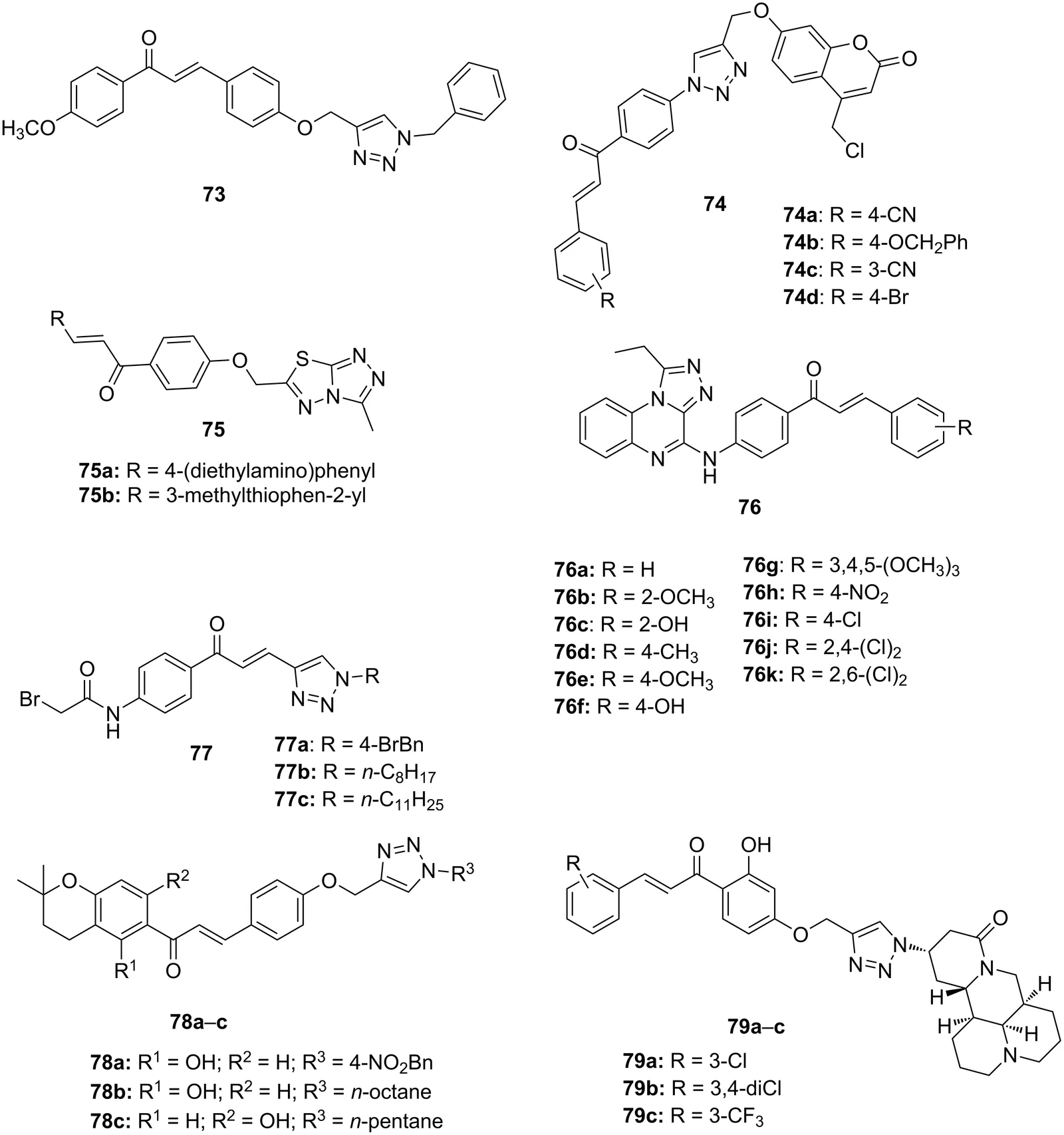
Structures of chalcone–triazole hybrids **73**–**79**.

Utilizing click chemistry under eco-friendly conditions, the chalcone–triazole hybrid **73** (Figure 14) was produced and assessed for its cytotoxicity against MCF-7, MIA PaCa-2, A549, and HepG2 cells [101]. The hybrid demonstrated the maximum effective cytotoxic activity, having IC_50_ values oscillating between 4–11 µM towards all tested cell lines. The mechanistic studies exposed that compound **73** brings apoptosis and causes G2/S phase cell cycle detention in MIA PaCa2 cells and, induces a mitochondrial potential loss in MIA PaCa-2 cells. Mallikarjun et al. (2023) evaluated the anticancer potential of novel coumarin-triazole-chalcone **74** against various human cancer cells, including HEK293 (human embryonic kidney cells), HeLa (cervix carcinoma), PANC1 (pancreatic cancer), HT1080 (fibrosarcoma), and A549 (lung cancer). Among synthesized hybrids, *para*-nitrile chalcone **74a** exhibited the most potent cytotoxicity with IC_50_ values ranging between 3.1 to 7.02 µg/mL against tested cell lines. In silico studies demonstrated that compound **74a** binds effectively to cancer-relevant molecular targets PI3K and AKT, showing specificity for AKT with binding interactions comparable to known AKT inhibitors [102].

The cytotoxic activity of 1,2,4-triazole–chalcone derivatives **75a** and **75b** (Figure 14) was assessed against several human cancer cell lines, including MCF-7, HeLa, A549, and SW620. Among these, compound **75a** demonstrated notable selectivity toward SW620 cells, exhibiting an IC_50_ value of 21.55 µM, while compound **75b** showed significant cytotoxicity against A549 cells, with an IC_50_ value of 25.58 µM [103]. A series of chalcone derivatives **76** containing a triazolo[4,3-*a*]quinoxaline scaffold (Figure 14) was investigated for their potential as chemotherapeutic agents, capable of simultaneously inhibiting tubulin polymerization and EGFR kinase activity. Among these, compound **76g** demonstrated the highest potency, attributed to its favorable interactions with the colchicine-binding site of tubulin. It exhibited IC_50_ values of 1.65, 3.61, and 8.58 µM against MCF-7, HCT-116, and HepG2 cancer cell lines, respectively [104]. The anticancer potential of 1,2,3-triazole–chalcone derivatives **77** (Figure 14) was evaluated against A549 cancer cell line, exhibiting IC_50_ values ranging from 8.67 to 11.62 µM. SAR studies indicated that the presence of a bromo substituent is critical for cytotoxic activity, while modifications on the heterocyclic ring led to a marked reduction in potency. Among the series, compounds **77a**, and **77c** demonstrated the highest antiproliferative effect, with IC_50_ values of 8.67 and 9.74 μM, respectively, approaching the efficacy of the reference chemotherapeutic agent, doxorubicin **(**IC_50_: 3.24 μM) [105]. The methoxy-bridged 1,2,3-triazole–chalcone derivatives **78a**–**c** (Figure 14) exhibit enhanced cytotoxic activity against A540 lung cancer cells (IC_50_ = 35.81–50.82 μM) compared to the standard chemotherapeutic agent doxorubicin (IC_50_: 69.33 μM), and demonstrated their potential as lead compound for lung cancer therapy [106]. The triazole–tethered chalcone–matrine hybrids **79a**–**c** (Figure 14) with electron-withdrawing groups exhibited enhanced antiproliferative activity (IC_50_** =** 5.01–12.72 μM) against A549 cells. The hybrid **79a** was showing the most potent cytotoxicity (IC_50_ = 5.01–7.31 μM) across evaluated cell lines, while showing minimal cytotoxicity against normal NIH/3T3 fibroblast cells (IC_50_ = 39.21 μM), indicating a favorable therapeutic index. Mechanistically, **79a** induces dose-dependent apoptosis in A549 cells [107].

**Figure 15:**
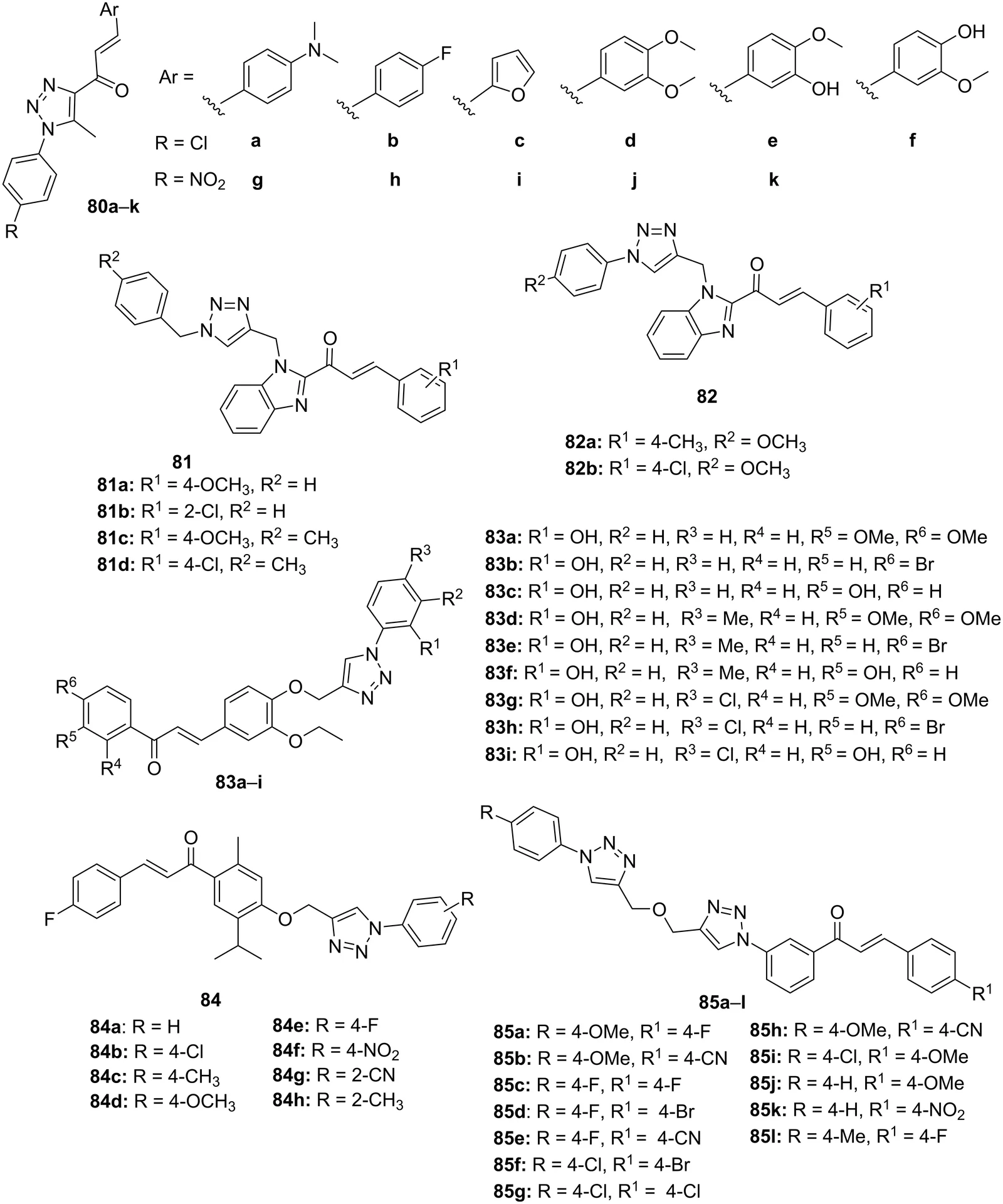
Structures of chalcone–triazole hybrids **80–85**.

The 1,2,3-triazole–chalcone derivatives **80a**–**k** (Figure 15) were assessed for cytotoxic activity against a diverse panel of 60 human cancer cell lines using the NCI-60 screening protocol. Among the synthesized candidates, **80d**, featuring methoxy groups at 3 and 4 position of the phenyl ring in the chalcone moiety, exhibited the most promising anticancer activity. At a concentration of 10 μM, **80d** achieved greater than 94% growth suppression in RPMI-8226 and SR leukemia cell lines, and demonstrated over 80% inhibition against M14 melanoma, K-562 leukemia, and MCF-7 breast cancer cells. Also, derivative **80d** displayed an IC_50_ value below 1 μM in six cancer cell lines, indicating high potency and selectivity. Compound **80d** induces G2/M phase cell cycle arrest and triggers apoptosis through the mitochondrial pathway [108]. Djemoui et al. (2020) elucidated the anticancer potential of the triazole–benzimidazole–chalcone hybrids **81** and **82** (Figure 15) against T47-D, MDA-MB-231 and PC-3 cancer cell lines. The presence of chlorine group on the phenyl ring of the chalcone led to a marked enhancement in cytotoxic activity. Additionally, the incorporation of a benzyl group on the triazole moiety was found to further potentiate the compound’s antiproliferative effects [109]. The chalcone hybrid tethered to a 1,2,3-triazole ring **83** (Figure 15) specifically **83d**, **83g**, and **83i** exhibited strong action towards MCF-7, MDA-MB-231 cancer cells, and HeLa cancer cells with lower IC_50_ value as compared to the standard drug, cisplatin. Also, these potent compounds exhibited low toxicity for normal breast epithelial cells (MCF-10A), indicating their selective behaviour [110]. The chalcone–thymol–triazole hybrids **84a–h** (Figure 15) were synthesized and subjected to in vitro antiproliferative evaluation towards four cancer cell lines: HT-1080, A-549, MCF-7 and MDA-MB-231. These hybrids showed cytotoxic effects, having IC_50_ concentrations ranging from 4.61 to 50 μM. Furthermore, hybrids bearing phenyl and cyano substituents on the triazole moiety particularly showed strong inhibition of HT-1080 cell proliferation, with IC_50_ values of 4.61 μM and 9.08 μM, respectively [111]. A recent study on chalcone-based derivatives **85a**–**l** containing two 1,2,3-triazole units (Figure 15) showed anticancer potential having IC_50_ values ranging from 44.7 to 86.2 μM, which is in contrast with the standard drug doxorubicin. The observed anticancer activity may be attributed to ERK2 kinase inhibition, which disrupts cell growth signaling and promotes cancer cell death [112].

### Pyridine–triazole hybrids

**Figure 16:**
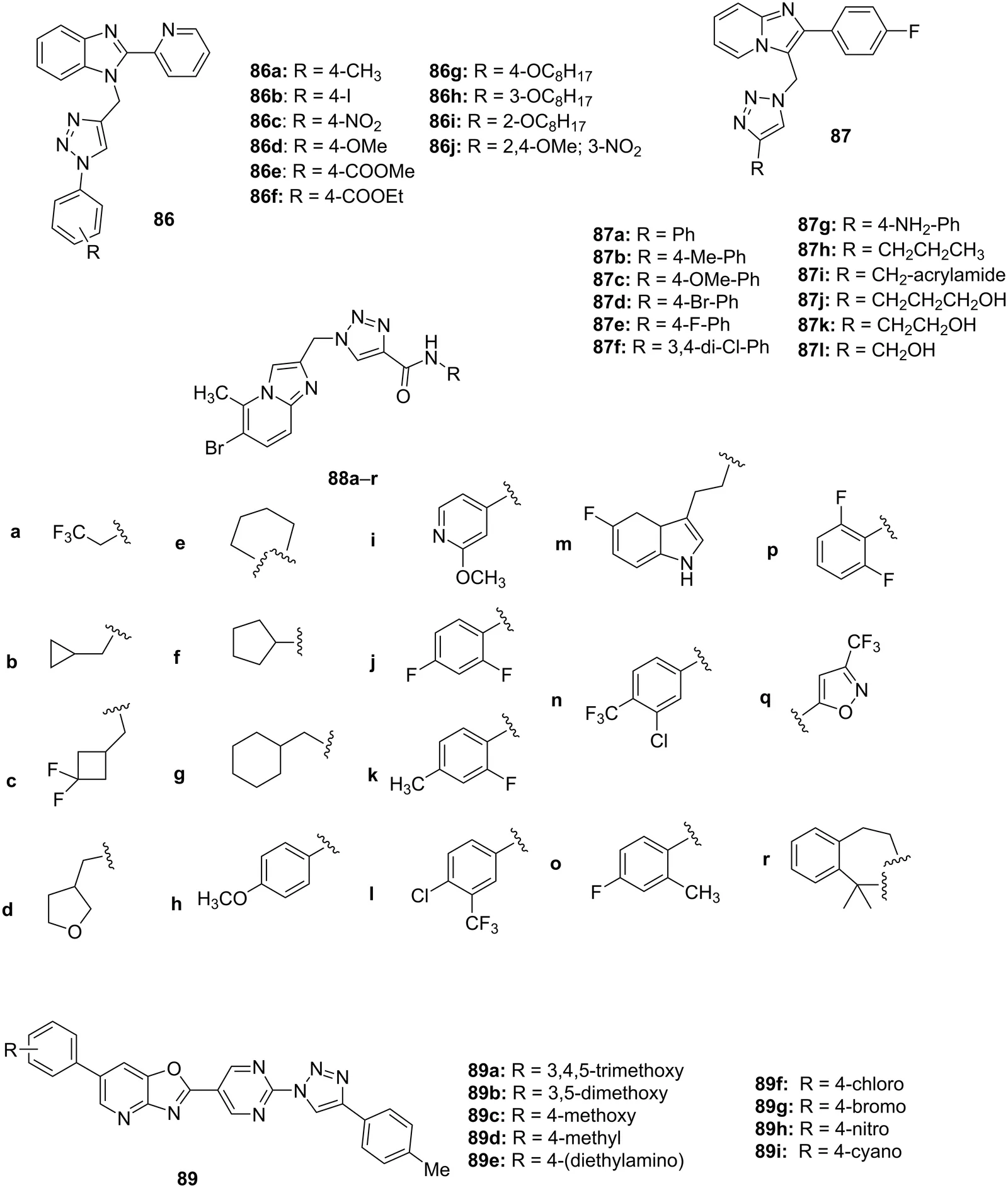
Structures of 1,2,3-triazole–pyridine derivatives **86**–**89**.

Pyridine scaffolds are widely recognized in medicinal chemistry for their crucial role in numerous clinically approved drugs, owing to their heteroaromatic nature and ability to intermingle to various biological targets such as kinases, enzymes, and nucleic acid [113–114]. Incorporation of a triazole moiety into these frameworks further enhances their pharmacological profile by improving metabolic stability, hydrogen bonding capacity, and target affinity. The novel 1,2,3-triazole–pyridine–benzo[*d*]imidazole hybrids **86** (Figure 16) were studied for their anticancer activity as prospective anticancer medications for the breast (MCF-7), colon (HCT116), human cell line (BJ1), and hepatocellular carcinoma (HepG2). Among synthesized candidates, **86g** showed superior cytotoxicity against HepG-2 (IC_50_ = 3.3 μM), hybrids **86c**, **86g**, and **86h** showed cytotoxicity towards MCF-7 with IC_50_ in between 3.0 to 3.5 μM. Against HCT-116, all synthesized hybrids showed better cytotoxic activity (IC_50_ = 5.2 μM to 6.2 μM) than doxorubicin (IC_50_ = 6.5 μM) [115]. A series of imidazo[1,2-*a*]pyridines-1,2,3-triazole conjugates **87** (Figure 16) were explored for cytotoxic effects across multiple cancer cell lines, including MCF-7, A549, HepG2, and T98G. The methoxy substituted derivatives exhibited the most potent antiproliferative activity and showed negligible cytotoxicity to normal human fibroblast cells (MRC-5), indicating a favorable therapeutic index. The mechanistic studies revealed a significant reduction in PARP protein levels in MCF-7 cells, suggested that apoptosis is mediated through PARP-dependent pathways [116]. The 1,2,3-triazoles linked via a methylene group to the imidazo[1,2-*a*]pyridine scaffold **88** (Figure 16) were tested towards two types of cancer cells (Hela and MCF-7) to determine their antitumor capabilities. Several of the synthesized compounds exhibited significant cytotoxicity, with IC_50_ values varying between 2.35 to 120.46 μM against the panel of tested cancer cell lines. Among them, compound **88d** demonstrated superior antiproliferative efficacy compared to cisplatin, exhibiting IC_50_ values of 10.89 μM against Hela and 2.35 μM against MCF-7 cell lines [117]. The oxazolo[4,5-*b*]pyridine conjugated 1,2,3-triazole hybrids **89a**–**i** (Figure 16) assessed for anticancer effects against PC3, DU-145, A549, and MCF-7 cancer cell lines. Although, the majority of the synthesized candidates exhibited good to moderate anticancer effects in comparison with the reference drug, etoposide, compounds **89a–e,** and **89i** demonstrated the strongest anticancer effects across all tested compounds. Notably, hybrid molecule **89a** showed superior anticancer activity against PC3, DU-145, A549, and MCF-7 cells with IC_50_ values of 0.01 ± 0.0074 μM, 0.043 ± 0.009 μM, 0.15 ± 0.038 μM, and 0.045 ± 0.0021 μM, respectively. These oxazolo[4,5-*b*]pyridine-based triazoles exhibited inhibitory activity against human dihydroorotate dehydrogenase (hDHODH), suggesting a possible mechanism underlying their anticancer effects [118].

**Figure 17:**
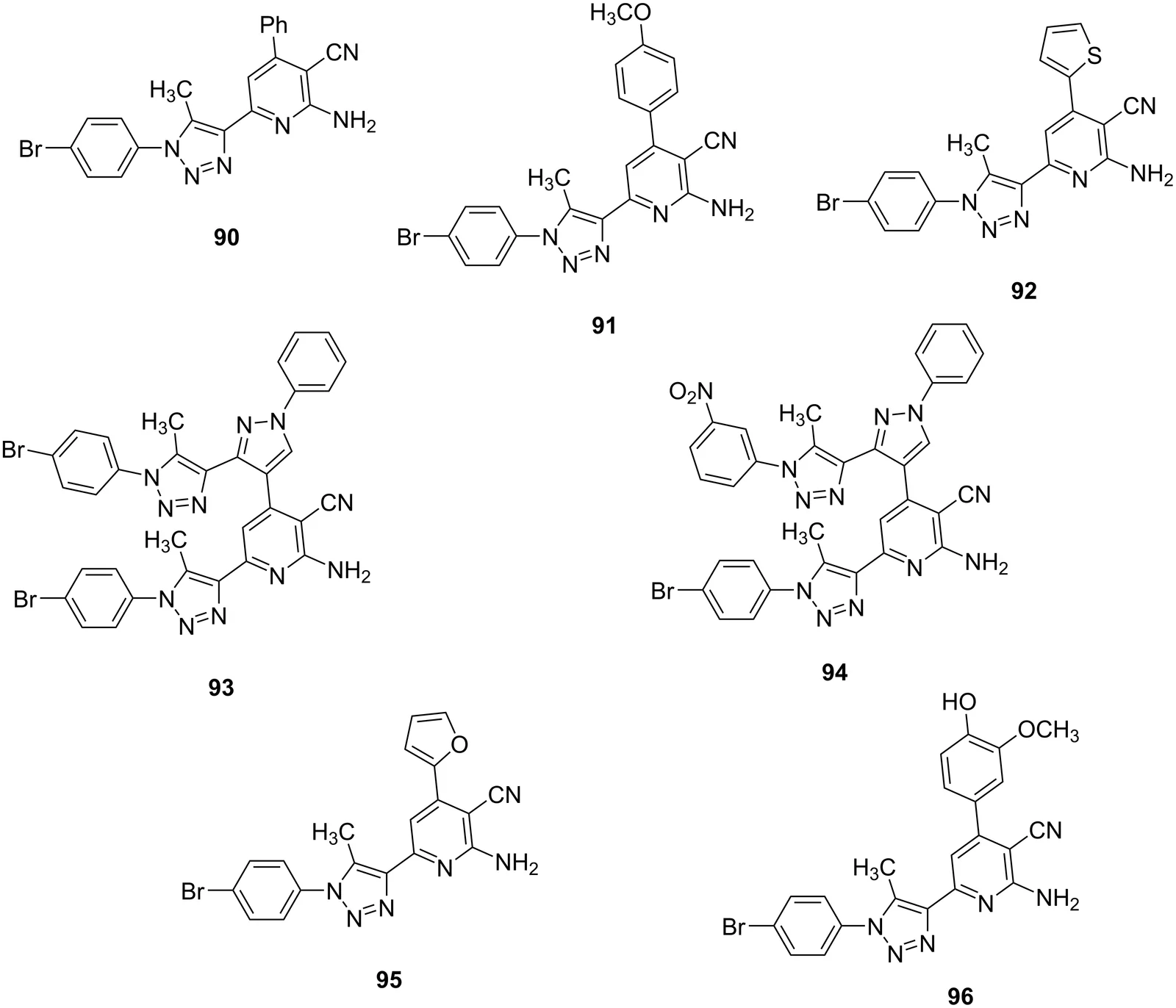
Structures of 1,2,3-triazolyl–3-cyanopyridine hybrids **90**–**96**.

**Figure 18:**
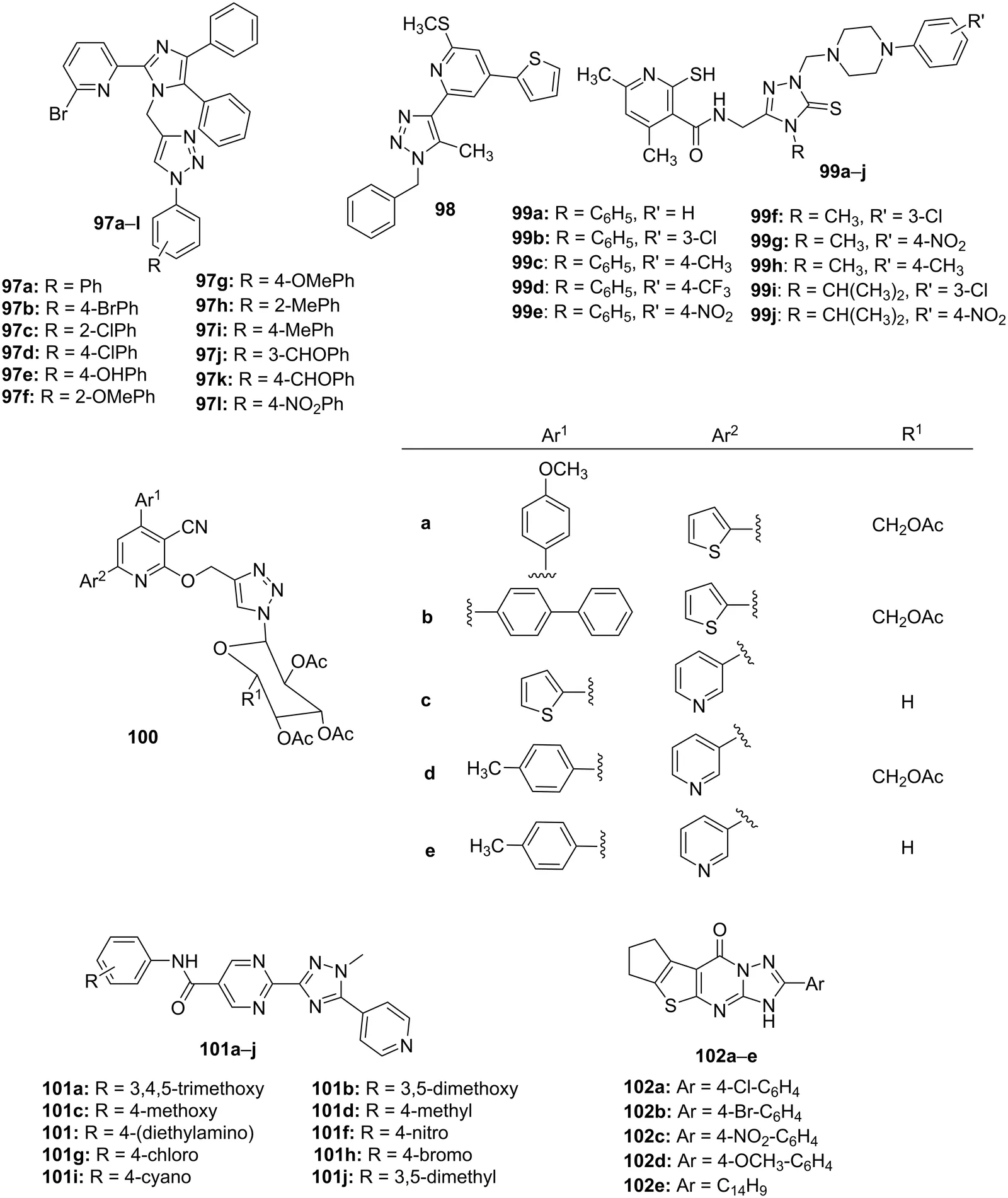
Structures of pyridine/pyrimidine–triazole hybrids **97**–**102**.

The estimation of the antihepatic cancer potential of 1,2,3-triazolyl–pyridine derivatives **90–96** (Figure 17) revealed notable cytotoxicity against human hepatoblastoma (HepG2) cells [119]. Most of the candidates exhibited promising anticancer activity, while hybrid molecule **93** (IC_50_ = 0.64 ± 0.15 μg/mL) and **94** (IC_50_ = 1.08 ± 0.95 μg/mL) demonstrated superior efficacy in comparison to the reference medicine, doxorubicin (IC_50_ = 3.56 ± 0.46 μg/mL). Further, these hybrids showed negligible cytotoxicity towards the normal BALAB/3T3 cell line, indicating a favorable selectivity profile and potential therapeutic window for hepatocellular carcinoma treatment. The anticancer potential of novel pyridine–imidazole–triazole hybrids **97a**–**l** (Figure 18) was evaluated against HT-1080 (fibrosarcoma) and Caco-2 (colorectal adenocarcinoma) cell lines. Among synthesized derivatives, compounds **97b**, **97c**, and **97f** displayed the strongest activity towards HT-1080, while compounds **97b** and **97c** exhibited significant cytotoxicity towards Caco-2 cells [120].

Thiophene-1,2,3-triazole–pyridine hybrid **98** (Figure 18) exhibited potent cytotoxicity towards A549 (IC_50_ = 0.68 μM), PC-3 (IC_50_ = 1.03 μM), and MDAMB-231 (IC_50_ = 0.88 μM). Molecular docking analysis revealed strong binding affinity of the compound to the ATP-binding site of human topoisomerase IIα [121]. The novel pyridine-1,2,4-triazoles **99a**–**j** tethered via an amide linker (Figure 18) were evaluated for their cytotoxic potential towards gastrointestinal cancer cell lines (EPG, Caco-2, LoVo, LoVo/Dx, HT-29), alongside normal colonic epithelial cells (CCD841 CoTr) for comparative assessment. Among the synthesized compounds, derivative **99c** featuring a phenyl substituent at the N-4 position and a 4-methylphenylpiperazine group at the N-2 of the 1,2,4-triazole ring demonstrated the most promising anticancer profile. It exhibited potent cytotoxicity against EPG and Caco-2 cancer cell lines while showing reduced toxicity towards normal epithelial cells, indicating a high degree of selectivity. Additionally, compound **99c** effectively inhibited P-glycoprotein in P-gp-expressing cell lines (HT-29, LoVo, and LoVo/Dx). Mechanistic studies further revealed that treatment with compound **99c** induced apoptosis in HT-29 cells via upregulation of caspase-3 and p53 protein levels [122]. The study on anticancer properties of a series of pyridine-1,2,3-triazole-*N*-glycoside derivatives **100** (Figure 18) found that compounds containing biphenyl or thienyl substituents on the phenyl ring (**100b** and **100c**) exhibited the most potent cytotoxic activity against A549, MCF-7, and PC3 cell lines. Molecular docking studies further revealed strong binding interactions of these derivatives within the EGFR active site, corroborating their proposed mode of action [123]. The novel pyridine–1,2,4-triazolyl–pyrimidine hybrids **101a**–**j** (Figure 18) were evaluated for cytotoxic potential against MCF-7, A549, Colo-205, and A2780 cancer cell lines. Most of the synthesized candidates demonstrated moderate to significant cytotoxicity relative to the reference drug, etoposide. However, compound **101a** showed particularly promising activity across the tested cell lines and showed IC_50_ values of 0.1 ± 0.075 μM, 0.26 ± 0.037 μM, 0.48 ± 0.048 μM, and 0.13 ± 0.023 μM on MCF-7, A549, Colo-205, and A2780 cell lines, respectively [124]. A series of novel thieno[2,3-*d*][1,2,4]triazolo[1,5-*a*]pyrimidine derivatives **102a**–**e** (Figure 18) was evaluated for cytotoxic activity against PC-3 (prostate), HCT-116 (colon), and MCF-7 (breast) cancer cell lines. Within the evaluated series, compounds **102b** and **102e** showed noticeable growth-inhibitory effects on the MCF-7 breast cancer cell line, with IC_50_ values of 19.4 ± 0.22 and 14.5 ± 0.30 μM, respectively, outperforming the standard chemotherapeutic agent, doxorubicin (IC_50_ = 40.0 ± 3.9 μM) [125].

### Miscellaneous

**Figure 19:**
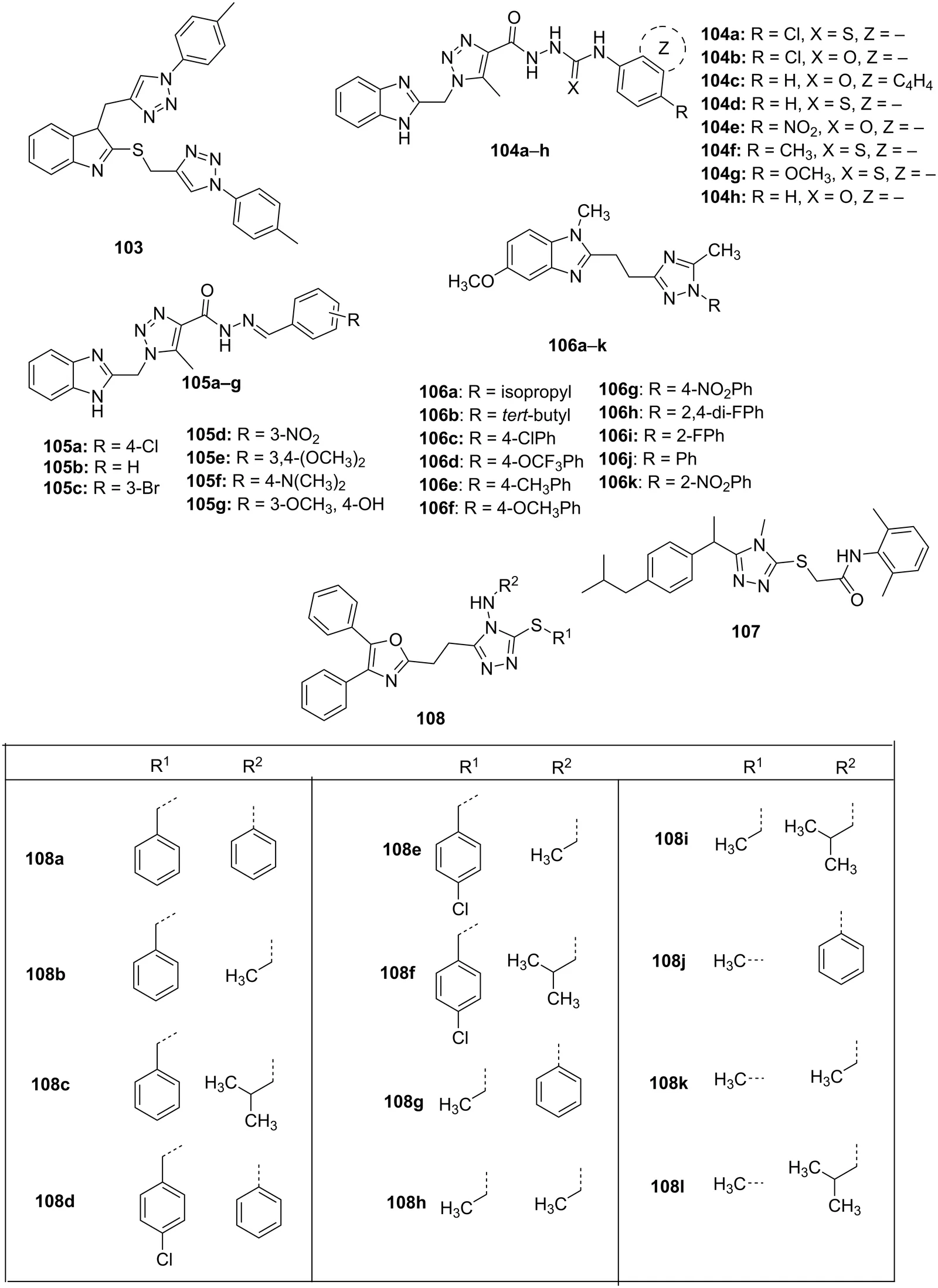
Structures of bistriazolyl-2-mercaptobenzimidazole **103**, benzimidazole-1,2,3-triazole conjugates **104**, **105**, benzimidazole–1,2,4-triazole derivative **106**, 1,2,4-triazole–acetamide hybrid **107**, and oxazol–1,2,4-triazole derivative **108**.

In addition to the above well-defined classes, several other triazole-based compounds with diverse scaffolds have also shown notable anticancer activity. These structurally varied hybrids often combine triazole with pharmacophores like benzimidazole, pyrimidine, or steroidal backbones. For instance, bistriazolyl derivative **103** (Figure 19) linked to a 2-mercaptobenzimidazole scaffold demonstrated significant cytotoxic activity against HepG2 and A549 cancer cells with IC_50_ values of 10.9 μM and 8.9 μM, respectively. Compound **103** exhibited aurora-A kinase inhibition that resulted in cell-cycle arrest and apoptosis induction [126]. Two different benzimidazole–triazole conjugates **104** and **105** (Figure 19) were reported as potent inhibitors targeting EGFR, VEGFR-2 and topoisomerase II. Among the synthesized hybrids, **104a** and **105g** emerged as lead candidates, showing significant cytotoxicity against HCT-116, MCF-7, HepG-2, and HeLa cancer cell lines. Compound **104a** demonstrated strong inhibition of EGFR with an IC_50_ value of 0.086 μM, comparable to gefitinib, along with moderate VEGFR-2 inhibition (IC_50_ = 0.109 μM) as compared to sorafenib, and enhanced topo II suppression (IC_50_ = 2.52 μM) relative to the standard anticancer agent doxorubicin (IC_50_ = 3.62 μM). Whereas compound **105g** showed a less potent profile across the three targets [127]. In a study by Chandrakar et al. (2024) benzimidazole-1,2,4-triazole derivatives **106a**–**k** (Figure 19) were tested for anticancer potential against various human cancer cell lines. Among the screened compounds, **106b** and **106g** demonstrated remarkable cytotoxicity on the HCA-7 colorectal cancer cell line, exhibiting IC_50_ values of 8.8 ± 0.9 μM and 9.2 ± 1.5 μM, respectively. Significant antiproliferative effects were also observed for **106d** and **106i** against MCF-7 cancer cells, with IC_50_ values of 8.3 ± 2.1 μM and 10.6 ± 1.2 μM, respectively. Also, **106d** and **106g** showed potent inhibitory activity against HT-29 colorectal cancer cells with IC_50_ values of 7.1 ± 0.9 and 7.7 ± 1.2 μM, respectively. Molecular docking revealed that **106d** interacts with the binding site of tyrosine phosphate (PDB ID: 1WCH), a critical enzyme involved in colorectal cancer signaling pathways [128]. The 1,2,4-triazole coupled acetamide derivative **107** (Figure 19), bearing two *ortho*-methyl substituents on the N-phenyl ring displayed antiproliferative activity on the HepG2 cell line with an IC_50_ value of 16.782 µg/mL. Molecular docking studies further revealed strong binding affinities of compound **107** towards c-kit tyrosine kinase and protein kinase B with values of −170.066 kcal/mol and −176.749 kcal/mol, respectively [129]. The antitumor potential of newly synthesized oxazol–1,2,4-triazole hybrids **108** (Figure 19) was evaluated against PC-93 and HBT-55 cell lines representing prostate and lung cancers, respectively. The study revealed that several compounds exhibited notable cytotoxic effects on the PC-93 cell lines, exhibited IC_50_ values varying from 13.12 to 24.80 μM, depending on the nature of the substituent. Furthermore, these derivatives demonstrated significant anticancer activity against the HBT-55 cell line, showing comparable or superior efficacy to the reference drug, doxorubicin (IC_50_ = 19.41 μM) [130].

**Figure 20:**
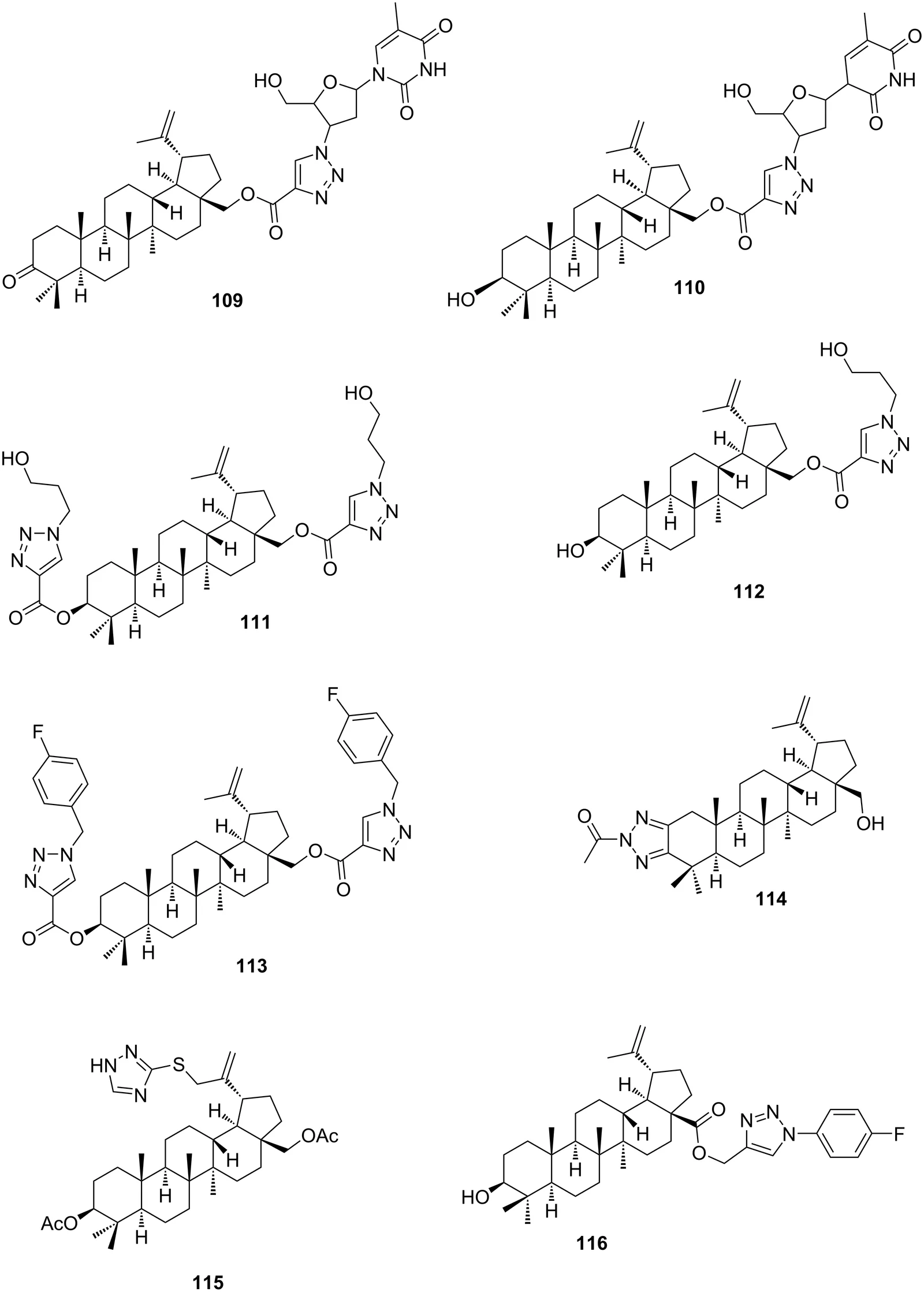
Structures of botulin-based triazole hybrids **109**–**116**.

A triazole derivative of betulone (**109**, Figure 20), exhibited exceptional cytotoxicity against the glioblastoma SNB-19 cell line, with an IC_50_ value of 0.17 μM, and demonstrated approximately five-fold higher efficacy than the standard drug cisplatin. The same derivative exhibited cytotoxic activity against amelanotic melanoma C-32 and exhibited an IC_50_ value of 0.81 μM [131]. This compound, therefore, represents a potential lead molecule for further development as an effective anticancer agent. The botulin–triazole derivatives **110** and **111** effectively suppressed the proliferation of SKOV-3 ovarian cancer cells by approximately 75% and 70%, respectively, at a concentration of 50 μg/mL. Despite exhibiting lower overall toxicity compared to betulin, their effective dosage indicated a strong influence in cell growth suppression. Compound **112** demonstrated pronounced cytotoxicity against T47D breast cancer cells, with an IC_50_ value of 1.3 μM, approximately 19 times more potent than cisplatin. Furthermore, the bistriazole derivative **113** exhibited exceptional cytotoxic activity against SNB-19, MCF-7, and T47D cell lines, with IC_50_ values of 0.08 μM, 0.09 μM, and 0.05 μM, respectively, underscoring its strong anticancer potential [132]. The *N*-acetyltriazole derivative betulin **114** showed significant cytotoxic activity against HCT 116, HEp-2, MS, and RD TE32 cancer cell lines, with IC_50_ values ranging from 2.3 to 7.5 µM [133]. Novel diacetylbetulin-1,2,4-triazole **115** exhibited anticancer activity against A375, MCF-7, and HT-29 cancer cell lines, with IC_50_ values ranging from 22.41 to 46.92 μM. Compound **115** causes apoptosis in tested cell lines, as confirmed by nuclear morphological alterations [134]. Triazole-tethered betulinic acid **116** demonstrated superior anticancer potential compared to betulinic acid, exhibiting IC_50_ values in the range of 5–7 µM against HL-60, MIA PaCa-2, PC-43, and A549 cell lines. In human leukemia HL-60 cells, it was found to induce apoptosis through both intrinsic and extrinsic pathways. This demonstrated the significance of triazole-tethered betulinic acids as promising candidates for anticancer drug development [135].

## Conclusion and Future Scope

Triazole-based hybrids have proven to be a multipurpose and promising class of heterocyclic compounds in anticancer drug development. Their unique ability to integrate diverse pharmacophores into a single molecular framework enables multi-target interactions and improved biological efficacy. This article emphasized the different classes of triazole hybrids such as coumarin–triazole, indole–triazole, chalcone–triazole, pyridine–triazoles etc., exhibiting cytotoxicity against a wide range of cancer cell lines. These hybrids often exhibit their anticancer effects via mechanisms such as kinase inhibition, topoisomerase inhibition, cell cycle arrest, and induction of apoptosis etc.

Though triazole-based hybrids exhibit encouraging in vitro cytotoxic potential; nevertheless, thorough in vivo studies remain relatively few. Prospective research ought to comprise:

- In vivo validation and translational studies through appropriate use of orthotopic and xenograft animal models, pharmacokinetic and pharmacodynamic profiling, risk evaluation and toxicological analysis and studying their bioavailability, metabolic pathways, and tissue dispersion.
- Use of proteolysis-targeting chimeras (PROTACs), a novel therapeutic approach for selectively degrading oncological proteins as triazole linkers provide excellent stability and biorthogonality. Nevertheless, traditional PROTAC synthesis is frequently time-consuming, including several synthetic stages, pre-assembled linkers, lengthy reaction durations, and low total yields. However, the one-pot synthetic approach is extremely effective, step-economic, easy to use, and environmentally benign, making it a useful platform for quickening PROTAC-based drug development [136].
- Incorporation of artificial intelligence and machine learning techniques for accelerating lead identification and optimization, predicting biological activity and toxicity, structure-based virtual screening & molecular docking and optimization of potency, selectivity, ADMET properties, and synthetic feasibility.
- Combining triazole hybrids with nanocarriers to lower off-target toxicity, increase the therapeutic index, targeted drug release, enhanced permeability and retention and improve their solubility in water.
- Adoption of a multi target drug design approach for synthesis of novel triazole hybrids that can modulate several biological pathways involving cellular proliferation, angiogenesis, apoptosis, metastasis, and drug resistance etc. These multi-target-directed triazole-based agonists would be safer, more efficient, and therapeutically transferable.

Despite the encouraging progress, challenges remain in translating these leads into clinical candidates. Overall, the structural diversity and therapeutic potential of triazole hybrids make them valuable candidates for further development as promising anticancer agents.

## Vitae


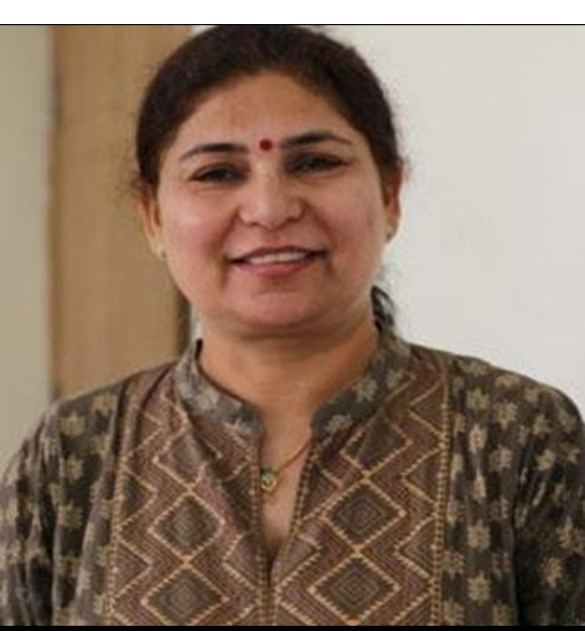


**Prof. (Dr.) Meena Bhandari:** Professor (Dr.) Meena Bhandari serves as the Dean, School of Basic and Applied Sciences, K.R. Mangalam University, and brings 27 years of distinguished teaching and research experience. She earned her Ph.D in Chemistry from IIT Delhi with specialization in calixarenes, along with an M.Phil. from Panjab University. Her research focuses on heterocyclic chemistry and green chemistry. She has successfully supervised four Ph.D scholars and is a recipient of the CSIR Fellowship and the AICTE Career Award for Young Teachers. Dr. Bhandari has published and presented 45 research papers in reputed SCIE, Scopus, and Web of Science indexed journals.


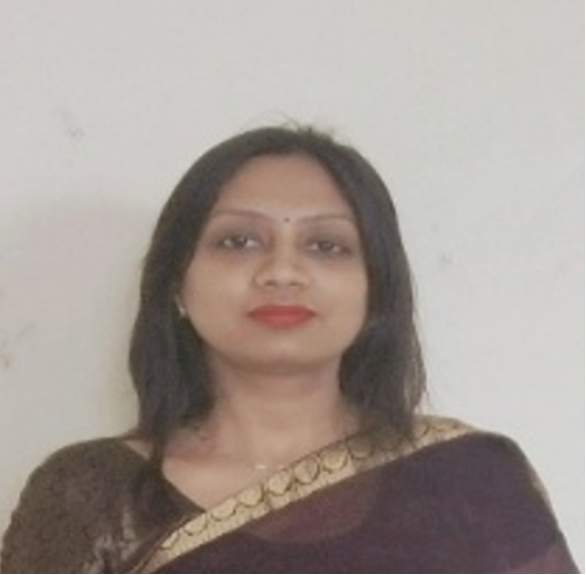


**Ms. Akshi Goyal:** Akshi Goyal is a Research Scholar in the Department of Chemistry, School of Basic and Applied Sciences at K.R. Mangalam University, Gurugram, Haryana. She completed her B.Sc. (2007) from Kurukshetra University, followed by M.Sc. in Chemistry (2009) from Kurukshetra University, where she earned a Gold Medal for academic excellence. Since 2019, she has been serving as an Assistant Professor of Chemistry at Government College for Girls, Manesar, Gurugram. Her academic and research interests focus on heterocyclic chemistry.


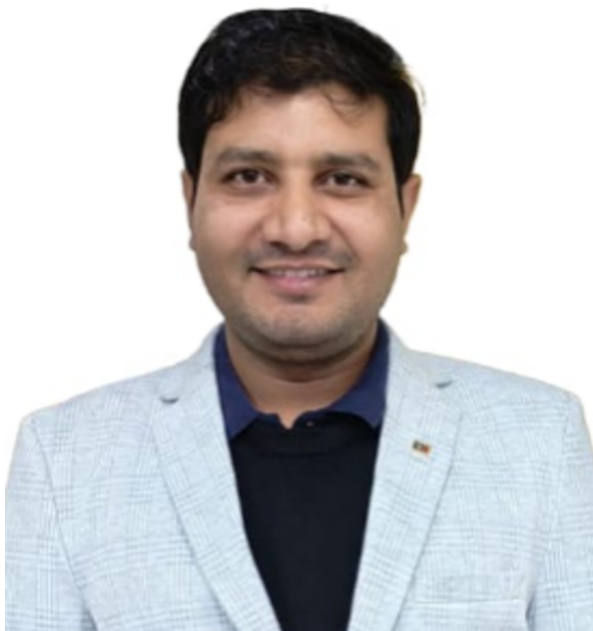


**Dr. Deepak Yadav:** Dr. Deepak Yadav is working as an Assistant Professor of Chemistry at Rajdhani College, University of Delhi. He obtained his Bachelor (2013) and Master (2015) from the University of Delhi. In 2015, he was awarded a research fellowship by CSIR-India. He earned his Ph.D degree in Chemistry in the year 2021 from the Central University of Haryana, Mahendergarh. He was a visiting fellow in the years 2019 and 2020 at Freie University of Berlin, Germany. His research work focuses on Chemistry of unsaturated sulfones, benzannulation reactions, and heterocyclic compounds. Dr. Yadav has published extensively in reputed journals and has also authored multiple book chapters in edited books.

## Data Availability

Data sharing is not applicable as no new data was generated or analyzed in this study.
